# Tulathromycin metaphylaxis increases nasopharyngeal isolation of multidrug resistant *Mannheimia haemolytica* in stocker heifers

**DOI:** 10.3389/fvets.2023.1256997

**Published:** 2023-11-20

**Authors:** William B. Crosby, Brandi B. Karisch, Lari M. Hiott, Lee J. Pinnell, Alexandra Pittman, Jonathan G. Frye, Charlene R. Jackson, John Dustin Loy, William B. Epperson, John Blanton, Sarah F. Capik, Paul S. Morley, Amelia R. Woolums

**Affiliations:** ^1^Department of Pathobiology and Population Medicine, College of Veterinary Medicine, Mississippi State University, Mississippi State, MS, United States; ^2^Department of Animal and Dairy Sciences, College of Agriculture and Life Sciences, Mississippi State University, Mississippi State, MS, United States; ^3^Poultry Microbiological Safety and Processing Research Unit, U.S. National Poultry Research Center, United States Department of Agriculture-Agricultural Research Service, Athens, GA, United States; ^4^VERO Program, School of Veterinary Medicine and Biomedical Sciences, Texas A&M University, Canyon, TX, United States; ^5^Nebraska Veterinary Diagnostic Center, School of Veterinary Medicine and Biomedical Sciences, University of Nebraska-Lincoln, Lincoln, NE, United States; ^6^Department of Animal Sciences, College of Agriculture, Purdue University, West Lafayette, IN, United States; ^7^Tumbleweed Veterinary Services, PLLC, Amarillo, TX, United States

**Keywords:** antimicrobial resistance, whole genome sequencing, antimicrobial susceptibility, bovine respiratory disease, beef production, bacterial culture

## Abstract

Bovine respiratory disease (BRD) is a leading cause of disease in feedlot and stocker calves with *Mannheimia haemolytica* (*MH*) as one of the most common etiologies. One of the most effective means of controlling BRD is through metaphylaxis, which involves administering antimicrobials to all animals at high risk of developing BRD. However, increasing prevalence of multidrug resistant (MDR) *MH* may reduce efficacy of metaphylaxis due to decreased susceptibility to drugs used for metaphylaxis. Primarily, this study aimed to determine the effect of tulathromycin metaphylaxis and subsequent BRD treatment on antimicrobial resistance (AMR) in *MH* isolated from stocker calves. Secondary objectives included evaluating the effect of metaphylaxis and treatment for BRD on animal health and comparing the genetic relationship of *MH* isolated. Crossbred beef heifers (*n* = 331, mean weight = 232, SD = 17.8 kg) at high risk for BRD were randomly assigned to receive tulathromycin metaphylaxis (META, *n* = 167) or not (NO META, *n* = 164). Nasopharyngeal swabs were collected for *MH* isolation, antimicrobial susceptibility testing and whole genome sequencing at arrival and 3 (WK3) and 10 (WK10) weeks later. Mixed-effects logistic regression was used to identify risk factors for isolation of *MH* and MDR *MH* (resistant to ≥3 antimicrobial drug classes) at 3 and 10 weeks, BRD morbidity, and crude mortality. Animals in the META group had higher odds of isolation of MDR *MH* at 3 weeks [OR (95% CI) = 13.08 (5–30.9), *p* < 0.0001] and 10 weeks [OR (95% CI) = 5.92 (1.34–26.14), *p* = 0.019] after arrival. There was no difference in risk of isolation of any *MH* (resistant or susceptible) between META and NO META groups at all timepoints. Animals in the NO META group had 3 times higher odds of being treated for BRD [WK3: OR (95% CI) = 3.07 (1.70–5.52), *p* = 0.0002; WK10: OR (95% CI) = 2.76 (1.59–4.80), *p* = 0.0002]. Antimicrobial resistance genes found within isolates were associated with integrative conjugative element (ICE) genes. Tulathromycin metaphylaxis increased risk of isolation of MDR *MH* and in this population, the increase in MDR *MH* appeared to be associated with ICE containing antimicrobial resistance genes for multiple antimicrobial classes. This may have important implications for future efficacy of antimicrobials for control and treatment of BRD.

## 1 Introduction

Bovine respiratory disease (BRD) is considered one of the costliest diseases to the beef cattle industry and has been estimated to cost the beef industry as much as $ 1 billion annually (1). BRD is thought to be caused by the interaction of microbial agents, host immunity, and environmental factors (2), but severe disease and death primarily results from fulminant bacterial bronchopneumonia (3). Bacteria that have been traditionally considered pathogens or pathobionts causing BRD include *Mannheimia haemolytica* (*MH*), *Pasteurella multocida*, *Histophilus somni*, and *Mycoplasma bovis*, with *MH* being the bacterial species most frequently isolated from the lungs of cattle that have died due to BRD (4). BRD diagnosis relies heavily on detection of clinical signs, including altered appetite, attitude, respiratory character, and fever. The methods have poor sensitivity and specificity leading to inaccuracies in identifying animals that would benefit from administration of antimicrobials for treatment (5). One of the most effective means of reducing BRD is metaphylaxis, the administration of antimicrobials to all animals at high risk of BRD at arrival to a production facility (6). Whereas drugs from multiple antimicrobial classes including macrolides, phenicols, cephalosporins, and fluoroquinolones are labeled for metaphylactic use, macrolide antimicrobials have been shown to be particularly effective (7, 8); however, there is increased scrutiny of metaphylaxis due to concerns of antimicrobial resistance (AMR) and the fact that it leads to administration of antimicrobials to some clinically healthy animals (9).

Isolation of AMR and multi-drug resistant (MDR) *MH* has become increasing common in the last decade. Klima et al. (10), reported that <10% of *MH* isolates collected in 2008–2009 from feedlot cattle in Alberta, Canada were resistant to any antimicrobials tested, and 1% showed MDR. Similarly, Noyes et al. (11) found that approximately 85% of *MH* isolated from NPS obtained from feedlot cattle were pansusceptible. However, Lubbers et al. (12), showed an increase in isolation of MDR *MH* when evaluating samples submitted to the Kansas State Veterinary Diagnostic Laboratory from 2009 to 2011, with 42% vs. 63% of isolates being considered MDR in 2009 and 2011, respectively. Numerous differences in these study designs exist, including sample collection site, geographic location, and others. In particular, Klima et al. and Noyes et al. evaluated *MH* isolated from the nasopharynx of cattle at arrival or exit from the feedlot, and Lubbers et al. evaluated isolates obtained from the lungs of cattle that had died due to BRD and were more likely to have been treated prior to sampling. Nevertheless, the report of Lubbers et al. brought attention to MDR *MH* associated with BRD. In 2012, an integrative conjugative element (ICE) was first described in *P. multocida* (13). This ICE (ICE*Pmu1*) contained multiple antimicrobial resistance genes (ARG), which conferred resistance to multiple classes of antimicrobials including aminoglycosides, beta-lactams, macrolides-lincosamides-streptogramins (MLS), phenicols, tetracyclines, and sulfonamides (14). ICE*Pmu1* was able to transfer *in vitro* to other bacterial species within and across genera (13). Since then, multiple ICE (ICE*Mh*-UGA1, -UGA2, and -UGA3 and ICE*Mh*1) have been identified within *MH* (15, 16). In 2016, Clawson et al. described 2 *MH* genotypes differentiated by more than 48,000 single nucleotide polymorphisms (SNPs) (17). In this study, only genotype 2 was associated with the lungs of cattle with BRD and ICE sequences, and a subtype within genotype 2 contained the majority of resistance genes found.

Stocker cattle are recently weaned beef breed cattle that are often managed on pasture for several weeks prior to transfer to feedlots to improve growth and immunocompetence. Multiple studies involving stocker calves have shown an increase in prevalence of MDR *MH* after administration of long-acting macrolides at arrival (18, 19). Woolums et al. (18) demonstrated an increase in MDR *MH* isolation at 3 weeks after arrival to a stocker operation in cattle that received tildipirosin on arrival. There was large genetic variation measured by pulse field gel electrophoresis in the MDR isolates, and AMR genes found had been previously associated with ICE (4). Snyder et al. also showed a sharp increase in isolation of MDR *MH* at 10–14 days compared to arrival and described 3 putative ICE sequences associated with resistance genes, in stocker calves that received tulathromycin metaphylaxis (15, 19). In both studies, all animals received macrolide metaphylaxis, and no animals not receiving metaphylaxis were tested, so the effect of metaphylaxis on AMR in *MH* in isolation from other characteristics of the cattle or environment could not be determined. Studies examining this question are somewhat conflicting. Tulathromycin metaphylaxis was identified as a risk factor for increased minimum inhibitory concentrations (MIC) to macrolides in *Pasteurellaceae* isolated from cattle after entry to feedlots in Canada (20). In contrast, Doster et al. (21) demonstrated that tulathromycin metaphylaxis had less effect than days on feed on the fecal microbiome and resistome in feedlot cattle (21). In 2016, DeDonder et al. (22) reported that there was a significant effect of animal source but not gamithromycin metaphylaxis on recovery of AMR *MH* in high-risk feedlot calves in Kansas. As these studies had differing results and used feedlot cattle, speculation on the effect of metaphylaxis on AMR in stocker calves is difficult, though one might expect similar results to DeDonder et al. (22).

The primary objective of this study was to investigate the effects of administration of macrolides at arrival on phenotypic and genetic markers of antimicrobial resistance of *MH* isolated from cattle on a stocker operation in the Southeastern U.S., and to evaluate the effect of metaphylaxis on *MH* isolation. Secondary objectives were to evaluate the effect of treatment for BRD on *MH* isolation and resistance, and effects of metaphylaxis on health measured by morbidity, mortality, and weight gain; evaluate the role of ICE and genotype on AMR in *MH*; and to evaluate the phylogenetic relationship among *MH* isolates.

## 2 Materials and methods

### 2.1 Animal use protocol and ethics statement

All animal handling, sampling, and treatment procedures were reviewed and approved by the Mississippi State University Institutional Animal Care and Use Committee prior to initiation of this study (Protocols: IACUC-18-529 and IACUC-21-558). When necessary, euthanasia via captive bolt and IV administration of KCl after sedation with 500 mg of xylazine (Rompun^®^, Dechra, Oakland Park, KS), was carried out by a trained veterinarian according to guidelines established the American Veterinary Medical Association (23).

### 2.2 Study design and animal population

This study utilized a complete randomized design with treatment group (TxGroup) as the experimental unit. This study was conducted in 4 Trials of approximately 80 beef type heifers in each Trial. There were 3 Trials started in late October (Fall 2019, 2020, and 2021), and one Trial started in mid-March (Spring 2021). Cattle for each Trial were purchased by an order buyer from regional auction markets near Starkville, MS (<6 h transport) over a 3-day period prior to arrival processing on study day 0 (d0), and animals were housed at an order buyer facility until calves were delivered to the H.H. Leveck Animal Research Center at Mississippi State University on the evening of d-2 or d-1. On the evening after arrival, animals were placed in a pasture with free access to water and hay.

**Figure 1:**
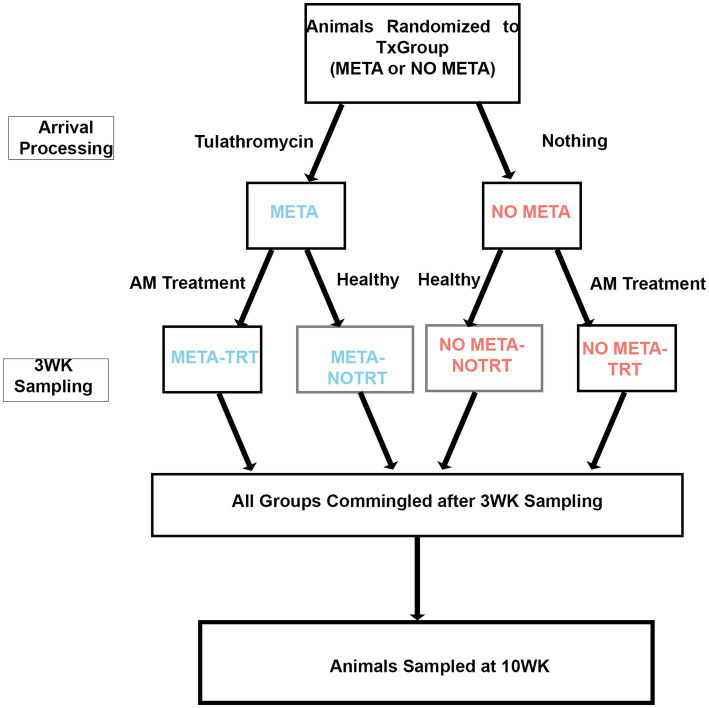
Study design diagram. TxGroup, treatment group; META, tulathromycin metaphylaxis; NO META, no tulathromycin metaphylaxis; TRT, treated with antimicrobials; NO TRT, not treated with antimicrobials; WK3, week 3; WK10, week 10.

Prior to each study, animal IDs were randomly assigned to receive tulathromycin metaphylaxis (META) or not (NO META) using the RAND function in Microsoft Excel for Mac (Microsoft, Redmond, WA, USA). After arrival processing, TxGroups (META or NO META) were separated into two 40-acre pastures with no fence-line contact and monitored daily for 20–21 days by trained pen riders on horseback, who were not blinded to TxGroup. Any animals requiring additional antimicrobial treatment (TRT) after receiving (or not) metaphyaxis were moved into separate pastures with no fence-line contact. Therefore, at the end of the first part of the study period (d.20 or d21) there were four groups of cattle: (1) META, (2) NO META, (3) META-TRT, and (4) NO META-TRT (Figure 1). Following d20 or d21 (WK3) sampling, animals were commingled into single pasture and monitoring intensity was decreased.

### 2.3 Animal handling

#### 2.3.1 Arrival processing

On d0, calves were brought to the processing barn and grouped in pens of approximately 20 while awaiting processing. For processing, animals were moved through a working chute, weighed, and had rectal temperature recorded. Additionally, all animals received a tag with individual ID in the left ear, and a color-coded tag to denote TxGroup (META or NO META) in the right ear. Previous identification of origin, such as farm tags or sale barn tags, were recorded and removed. Cattle were dewormed with oral fenbendazole (Safe-guard^®^, Merck Animal Health, Madison, NJ) and injectable doramectin (Dectomax^®^, Zoetis, Kalamazoo, MI). All animals received a vaccine for common respiratory viruses (Pyramid 5^®^, Boehringer Ingelheim Vetmedica, Inc., St. Joseph, MO) and clostridial vaccine (Bovilis^®^ Vision^®^ 7 with SPUR^®^, Merck Animal Health, Omaha, NE). Animals were revaccinated with both clostridial and respiratory vaccines on either d20 or d21 depending on weather and chute availability. All vaccines and anthelminthics were administered according to label directions, and all injections were given subcutaneously if indicated by the label (Datasheet S1). Ear notch samples were obtained on d0 for testing to detect persistent infection with bovine viral diarrhea (BVD-PI). Ear notches were kept on ice and delivered as soon as possible to the Mississippi Veterinary Research and Diagnostic Laboratory in Pearl, MS where they were tested via antigen capture ELISA. For all Trials except Spring 2021, samples were delivered overnight and tested the next day. In Spring 2021, d0 occurred on a Friday, so samples were frozen until shipping on Monday morning. Any animals found to be BVD-PI positive were removed from the study and euthanized as soon as results were received.

After sample collection, discussed below, META animals received 2.5 mg/kg tulathromycin (Draxxin^®^, Zoetis, Kalamazoo, MI) subcutaneously in the right side of the neck. NO META cattle received no antimicrobials on d0, but all other sampling and processing were the same between TxGroup.

#### 2.3.2 Animal health monitoring and treatment

Cattle in all pens were monitored daily for signs of illness including BRD. Any animals suspected to have BRD were given a clinical BRD score based on attitude, appetite, and respiration, as described previously (24), (Datasheet S2). Briefly, animals were given a score from 1–4 as follows: 1 = mild BRD, 2 = moderate BRD, 3 = severe disease, and 4 = moribund or near death. Due to the prolonged presence of tulathromycin in the pulmonary epithelial lining fluid, META animals with a BRD score 1 or 2 were not eligible for treatment until d8 of the study; all animals scoring 3 or 4 were eligible for treatment on d1. Animals eligible for BRD treatment were moved to a treatment chute and treated based on rectal temperature and BRD score (18) (Datasheet S3). Briefly, animals with a BRD score of 1 or 2 and a rectal temperature ≥104°F (40°C) were treated with antimicrobials. Animal with a score of 3 or 4 were treated regardless of rectal temperature. Animals were treated first with 6.6 mg/kg ceftiofur CFA (Exede^®^, Zoetis, Kalamazoo, MI), followed by 40 mg/kg florfenicol (Nuflor^®^, Merck & Co., Rahway, NJ), and 19.8 mg/kg oxytetracycline (Noromycin 300^®^,Norbrook, Lenexa,KS) for 2nd and 3rd BRD treatments if necessary, respectively, if there was no response to previous treatment (Datasheet S3). Post-treatment intervals (PTI) were 7 days for ceftiofur, 4 days for florfenicol, and 2 days for oxytetracycline. Animals were not eligible for another BRD treatment within their PTI. Animals not responding to antimicrobial therapy after treatment 3 were examined by a licensed veterinarian (WBC/ARW) and managed individually.

### 2.4 Sample collection and handling

#### 2.4.1 Nasopharyngeal samples

On d0, all animals had 4 deep nasopharyngeal samples (NPS) obtained with 75-cm double-guarded uterine culture swabs as described previously (25). Briefly, the external nares were cleaned with a clean disposable paper towel. Next, a double-guarded swab (E9-5200, Continental Plastic, Delavan, WI) was advanced through the ventral meatus to approximately the level of the medial canthus. The inner guard was advanced, followed by the swab, and the swab was turned 3–5 full rotations. The swab was then removed while leaving the guards in place and examined for mucus indicating a successful sample. The swab was then placed in a transport tube containing Modified Amies Transport Media (Starplex Scientific Corporation, St. Louis, MO), and the swab handle was cut with scissors cleaned with 70% isopropyl alcohol. To reduce the trauma of passing the double-guarded swabs in the same nostril multiple times, for the second swab a clean swab was carefully removed from its package and inserted into the guard passed for the first swab which was maintained in place in the nasal passage. The swab was advanced, and sample collected as before, and the swab and guard were removed. The second swab was placed in a 2 mL cryovial, and the handle cut. The sampling was repeated in the right nostril, and the first swab from each nostril was paired in one Modified Amies Transport Media tube for bacterial culture, and the second swab from each nostril was paired in a single 2 mL cryovial for DNA extraction and molecular analysis to be discussed elsewhere. Each tube was labeled with animal identification and date and placed on ice until transportation to the laboratory for processing. Swabs were also collected on d20 or d21 (WK3) and d69 or d70 (WK10), depending on weather and availability of working facilities, and before administration of antimicrobials for any animals requiring antimicrobial therapy for illness. After completion of sampling (approximately 4–6 h), samples were transported to the laboratory (~10 min) for further processing.

#### 2.4.2 *Mannheimia haemolytica* culture and identification

The identity of suspected *MH* colonies isolated from culture was confirmed as previously described (26). Briefly, paired swabs from each animal were directly streaked onto a plate of tryptic soy agar (TSA) with 5% sheep blood (BAP; Remel, Lenexa, KS). Plates were incubated at 37°C and supplemented with 5% CO_2_. Colonies with growth consistent with *MH* (2–3 mm, round, raised, light-gray, smooth, shiny colonies with faint β hemolysis) underwent preliminary biochemical tests (catalase, oxidase, and indole). If preliminary biochemical tests were consistent with *MH* (catalase-positive, oxidase-positive, and indole-negative), a single colony was selected arbitrarily and subcultured onto a new BAP and returned to the incubator at the above conditions. After 24 h, subcultures were monitored for colony phenotype and biochemical tests consistent with *MH*, as above. If suspect *MH* were present, 4–5 distinct colonies were arbitrarily selected from the subplate and suspended into 1.2 mL cryovials containing 1 mL of brain heart infusion (BHI) broth (B-D, Franklin Lakes, NJ) and 30% glycerol (Thermofisher, Waltham, MA). These isolates were stored at −80°C until overnight transport on dry ice to the USDA-ARS U.S. National Poultry Research Center (USNPRC) in Athens, GA for antimicrobial susceptibility testing (AST) and whole genome sequencing (WGS). Samples from the Fall 2019 Trial were kept at −80°C for 10 weeks before shipping. Samples from all other Trials were shipped at the completion of the Fall 2021 Trial and shipped in March of 2022.

The same loop used to pick suspect *MH* colonies was then used to streak another BAP which was then incubated as described above for 18 h then shipped overnight on ice to University of Nebraska-Lincoln Veterinary Diagnostic Center (UNL-VDC) to confirm identity. Primary plates with no suspected *MH* growth after 72 h were considered culture-negative for *M. haemolytica.*

At UNL-VDC, a single colony from the shipped plate was subcultured overnight on BAP to ensure pure growth. Matrix assisted laser desorption-ionization time-of-flight mass spectroscopy (MALDI-TOF) was used to confirm *MH* identity as well as MALDI-TOF biomarker-based genotyping of *MH* isolates (27).

#### 2.4.3 Antimicrobial susceptibility testing of *Mannheimia haemolytica* isolates

*Mannheimia haemolytica* isolates were received at USNPRC in cryovials. Each sample was streaked onto a BAP (BAP1) and incubated at 36°C for 18–24 h. One suspect *MH* colony was then subcultured onto a new BAP (BAP2) and streaked for isolation, and BAP2 were then incubated for 18–24 h and *MH* colonies were frozen at −80°C in BHI broth with 30% glycerol. Well-isolated colonies from BAP2 were used for AST following the National Antimicrobial Resistance Monitoring System (NARMS) protocol for susceptibility testing of Gram-negative bacterial species (28) using semi-automated broth microdilution via the Sensititre system (ThermoFisher, Waltham, MA) and the bovine/porcine panel containing gamithromycin and tildipirosin (BOPO7F Vet AST Plate, ThermoFisher, Waltham, MA). Results were interpreted according to breakpoints for *MH* in BRD from the Clinical and Laboratory Standards Institute (CLSI) (29). Isolates were characterized as multidrug resistant (MDR) if they were not susceptible to ≥3 antimicrobial drug classes (30). Because the concentration range for ampicillin on the BOPO7 plate does not include CLSI breakpoints, only MICs were recorded, and ampicillin resistance classification was not included in determination of isolates as MDR. To visualize relationship between metaphylaxis on phenotypic resistance, heatmaps were constructed using the ‘pheatmap’ package in R version 4.0.3 (31, 32), ‘not interpretable’ coded as ‘0’, ‘susceptible’ coded as ‘1’, and ‘not susceptible’ coded as ‘2’ in the heatmap. The heatmap was annotated with the following metadata: (1) TxGroup, (2) Trial, (3) reason for sampling, (4) antimicrobial treatment before sampling, (5) MALDI biomarker genotype, and (6) presence of ICE.

#### 2.4.4 Whole genome sequencing of *Mannheimia haemolytica* isolates

From a fresh BAP2 plate, a single, well-isolated colony was suspended in 9 mL of BHI broth and incubated overnight with shaking at 37°C. DNA was extracted using the DNEasy Blood and Tissue Kit (Qiagen, Germantown, MD) following manufacturer protocol (33). DNA quality was assessed using a Nanodrop 2000 Spectrophotometer (ThermoFisher Scientific, Wilmington, DE), and DNA concentration was determined using the Qubit 4 Fluorometer (ThermoFisher Scientific, Wilmington, DE). DNA libraries were prepared using the Illumina Nextera XT DNA library kit following manufacturer directions with Set A primers (34). Sequencing was performed using Illumina MiSeq with the MiSeq Reagent Kit v2, 500 cycle kit (MS-102-2003, Illumina, San Diego, CA). Fastq files were then assembled into draft genomes using the a5 MiSeq assembly pipeline (35–36), an automated *de novo* assembly pipeline that uses ‘Trimmoatic’ (37) and ‘SGA’ (38) for trimming and error correcting, ‘IDBA-UD’ for contig assembly, and ‘SSPACE’ for scaffolding (39). Whole genome assemblies were uploaded to NCBI Genbank (PRJNA942944: SAMN33700459- SAMN33700763) and annotated using the Prokaryotic Gene Annotation Pipeline (PGAP) (40–42). Sequence reads were submitted to NCBI Sequence Read Archive (PRJNA942944: SAMN33700459- SAMN33700763).

##### 2.4.4.1 Determination of resistance genes

Antimicrobial resistance genes present in the draft genomes were identified from the MEGARes v. 3.0 database (43) using blastn (BLAST +2.12.0) (44), with percent identity >80% and e-value <10^−7^ as cutoffs for hits. Only sequences covering >40% of the resistance gene were considered present. Since some ARGs require a single nucleotide polymorphism (SNP) to confer resistance, these SNPs needed to be confirmed in the draft genomes. Files in the default BLAST output format was converted to ‘.sam’ files using blast2sam in samtools (45), and the ‘.sam’ files were used for SNP confirmation using AmrPlusPlus_SNP (43). To visualize the relationship between metaphylaxis and ARGs found, heatmaps were constructed using the ‘pheatmap’ package in R version 4.0.3 (32), absence coded as ‘0’ in the heatmap and presence recorded as ‘1’. Isolates were considered genetically MDR if they contained genes that conferred resistance to ≥3 antimicrobial classes.

##### 2.4.4.2 Identification of integrative and conjugative elements

Genes associated with ICE previously found in *Pasteurellacea* (Table S1) (13, 16) were identified in the assembled genomes using blastn (BLAST+ 2.12.0). Only alignments with 95% identity and with 100% coverage of the ICE gene were considered as having the gene present in the assembly (15). Each isolate was considered to be positive for ICE if it had: (1) at least one of *int1* or *int2*, AND (2) at least one of *rel1* or *rel2*, AND (3) at least one of *traC-, traD-, or traG-*like genes (Table S1).

##### 2.4.4.3 Phylogenic analysis of *MH* isolates

To determine the genetic relationship of *MH* isolated in this study, a maximum likelihood tree of the pangenome was constructed using IQTree with Model Finder (46). Genomes of *MH* isolated in this study were isolated with Prokka (47), and the resulting ‘.gbk’ files were used to determine homologous regions using get_homologues (48, 49) and cluster homologous regions using OrthoMCL v 1.4 (50). *Mannheimia haemolytica* USDA-ARS-USMARC-191 (GenBank: CP023044.1) was included for reference. A pangenome matrix was constructed of clustered orthologs using ‘compare clusters’ within get_homologues (49), and the reduced binary FASTA was input to IQ-Tree to generate a maximum likelihood tree. The optimal substitution model (JC2 + FQ + R8) was chosen using ModelFinder (46), and bootstrapping of 1,000 iterations was done with UFBoot for ultrafast bootstrapping (51). The resulting tree was visualized with FigTree v 1.4.4 (52) and midpoint rooted. The midpoint rooted tree was annotated with important metadata using EvolViewerv3 (53).

#### 2.4.5 Comparison of genotypic and phenotypic resistance

To illustrate the relationship of genetic and phenotypic antimicrobial resistance, a concordance table was made. Comparisons were made at the class level for antimicrobials and resistance genes. For genetic resistance, isolates were considered positive for resistance to a class if they had at least one copy of a gene conferring resistance to that class, and isolates were considered MDR if they contained resistance genes to ≥3 antimicrobial classes. For phenotypic resistance, isolates were considered resistant if they were classified as intermediate or resistant to any antimicrobial of that class (30).

### 2.5 Other sampling

Weights were recorded from d0 through d21 at weekly intervals, and at d70. Because these calves were included in additional research investigations, up to 45 mL of blood was collected via jugular venipuncture at various time points. Though this varied among Trials 1–4, within any Trial animals across TxGroup were sampled similarly.

### 2.6 Statistical analysis

#### 2.6.1 Comparison of treatment group at arrival

Summary statistics of weight, isolation of *MH*, MALDI genotype of *MH* isolated, and MDR status of *MH* isolated at arrival were calculated using the ‘stats’ package in R version 4.0.3 (31). Comparisons were made between the TxGroup using a *χ*^2^-test for isolation of *MH* and isolation of MDR *MH* at arrival; the same comparisons were made among Trials using a Fisher’s Pairwise Exact *χ*^2^-test, using the ‘rstatix’ and ‘stats’ packages in R (31, 54).

#### 2.6.2 Descriptive statistics of animal health data and *MH* isolation

Summary statistics of averaged daily gain (ADG), crude morbidity (number of animals receiving treatment for any reason among the entire population), BRD morbidity (number of animals requiring at least one antimicrobial treatment for BRD among the entire population), crude mortality (number of animals who died or were euthanized for any reason among the entire population), *MH* isolation, and MDR *MH* isolation using ‘rstatix’ and ‘stats’ packages in R version 4.0.3 (31, 54). Comparisons of those outcomes between TxGroup were made using Wilcoxon Sum Rank Test for continuous data (ADG) and *χ*^2^ test for categorical data (crude and BRD morbidity, crude mortality, *MH* isolation, and MDR *MH* isolation), with a critical *α* of 0.05. The same comparisons were made between TxGroups within each Trial. Comparisons among Trials grouped by TxGroup were made using Kruskal-Wallis Analysis of Variance on Ranks for continuous data and Fisher’s exact test for categorical data. If Kruskal-Wallis or Fisher’s exact test were found to be significant (*p*≤0.05), *post hoc* analysis was performed with a Dunn Test or Pairwise Fisher’s Exact Test, respectively.

#### 2.6.3 Effect of metaphylaxis on *MH* isolation and antimicrobial resistance and animal health at WK3 and WK10

Mixed effect logistic models were constructed using the ‘lme4’ (55) package in R v. 4.0.3 with the outcomes of: (1) isolation of *MH* at 3 and 10 weeks, (2) isolation of MDR *MH* at 3 and 10 weeks, (3) isolation of ICE positive *MH* at 3 and 10 weeks, (4) isolation of MALDI genotype 2 *MH* at 3 and 10 weeks, (5) treatment for BRD before 3 and 10 weeks, and (6) death before 3 and 10 weeks. For all models, TxGroup was included in the model, and Trial (F19, F20, S21, F21) was included as a random effect. Other variables included for testing in the multivariable model were: (1) isolation of *MH* at a previous time point, (2) isolation of MDR *MH*, Genotype 2 *MH*, or isolation of *MH* containing ICE at a previous timepoint, (3) previous treatment for BRD, or (4) difference from median arrival weight (Table S2). Univariable models were constructed with Trial as a random effect. Any variables that had an effect (*p* ≤ 0.2) on the outcome variable of interest were considered for inclusion in the final, multiple variable model using forward manual selection and an α of 0.05. Variables with a significant effect (*p* ≤ 0.05) were kept in the final model, and interactions were tested. If significant interactions were found, pairwise comparisons of least squares means differences among groups were made using the ‘lmerTest’ package in R (56).

Linear mixed models with the outcomes of average daily gains at 3 and 10 weeks were constructed similarly.

#### 2.6.4 Comparison of phenotypic and genetic classification of MDR *MH*

Classification of isolates as MDR by culture and susceptibility testing or presence of antimicrobial resistance genes was compared using McNemar’s *χ*^2^ Test.

## 3 Results

### 3.1 Animal population

A total of 335 animals were sampled at arrival over the 4 Trials. Three (3) animals were removed from the study prior to the WK3 sampling timepoint, and 1 animal was removed after WK3 but prior to WK10. One (1) of the removals was due to being BVD-PI positive (NO META, Spring 2021), and the other 3 were due to potential exposure to animals outside of the study when they were taken to the Mississippi State University-College of Veterinary Medicine Animal Health Center for evaluation of severe lameness. Measurements obtained from these animals after removal were not used in statistical analyses.

### 3.2 Isolation of *MH* and WGS assembly statistics

A total of 305 *MH* isolates were identified and underwent AST and WGS. Only one isolate per animal per sampling day was collected and used for analysis. Full antimicrobial susceptibility data, antimicrobial resistance gene counts, and metadata can be found in Tables S3–S5.

There was a median genome size of 2.62 Mb (range = 2.47–2.72 Mb) and a median coverage of 95.8X (range = 45.3–219.6X). Full assembly statistics can be found in Table S6.

### 3.3 Identification of ARGs

All resistance genes found were ‘Drug’ Type resistance genes, versus metal or biocide resistance determinants (43). Resistance genes were found that confer resistance to aminoglycosides (*aph(3′)* and *aph(6)*), beta-lactams (*bla*_ROB_ and *bla*_OXA_), macrolides-lincosamides-streptogramins (MLS) (*erm(42)*, *msr*E, and MLS23S group of genes), phenicols (*floR*), tetracyclines (*tetH* and *tetR*), and elfamycins (TUFAB) (Table S3). Genes that required SNP confirmation were *tetR*, TUFAB, and the MLS23S gene group. Elfamycins are not used in veterinary medicine, so TUFAB was not used in statistical analysis or in class counts for MDR determination. The MLS23S SNP is not in the AMRPlusPlus_SNPVerification database, so its presence could not be confirmed, and it was not used in further analyses.

#### 3.3.1 Association of phenotypic susceptibility and identification of antimicrobial resistance genes

As there was no difference between AST and ARG in classification of isolates as MDR (Table S5, McNemar’s *χ*^2^ Test, *p* > 0.05), AST results were used to define MDR for further analysis. For all antimicrobial classes in which resistance was identified among any isolate by phenotype and genotype, there was greater than 80% agreement for classification of resistance by AST or ARG (Table S7).

### 3.4 Isolation of *MH* at arrival

**Figure 2:**
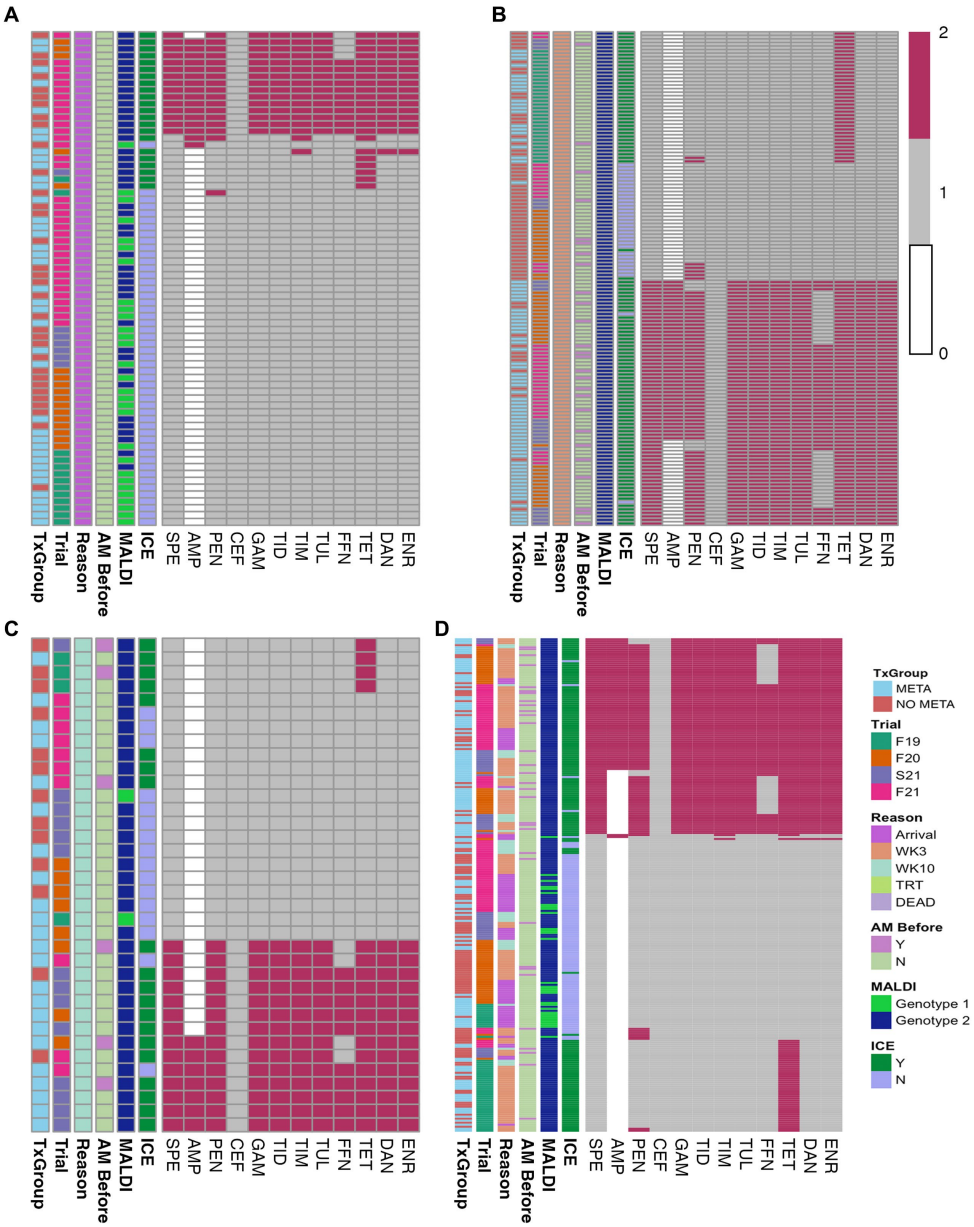
Heatmaps of *in vitro* susceptibility of isolated *MH*. **(A)** Arrival isolates, **(B)** WK3 isolates, **(C)** WK10 isolates, and **(D)** all isolates combined. TxGroup, treatment group; AM Before, treated with antimicrobial before isolation; MALDI, MALDI biomarker genotype; F19, Fall 2019; F20, Fall 2020; S21, Spring 2021; F21, Fall 2021; META, tulathromycin metaphylaxis; NO META, no tulathromycin metaphylaxis; MLS, macrolide-lincosamide-streptogrammin; ICE, integrative conjugative element; Y, Yes; N, No; WK3, week 3; WK10, week 10. Key: 0 = Not interpretable, 1 = susceptible, 2 = not susceptible.

Of the 335 animals sampled, *MH* isolation was identified among 72 at arrival (Figure 2A and Table 1). Most of these isolates (50/72) did not contain ICE (Figure 2A; Table S4). Forty percent (29/72) of arrival isolates, were classified as genotype 1 by MALDI-TOF (Figure 2A; Table S4).

**Table 1:** *Mannheimia haemolytica* isolation and weight of animals at arrival.

Trial	TxGroup (n*)	Arrival *MH* culture n (%)	MDR *MH* n (%)	Median arrival weight (95% CI) (kg)
Fall 2019	META (41)	10 (24.4)^a^	0 (0)	228 (215–242)
	NO META (41)	2 (4.9)^b^	0 (0)	230 (219–241)
	All (82)	12 (14.6)^c^	0 (0)^e^	229 (217–241)
Fall 2020	META (42)	8 (19.0)	3 (7.1)	230 (218–238)
	NO META (41)	9 (22.0)	1 (2.4)	230 (218–239)
	All (83)	17 (20.5)^c^	4 (4.8)^ef^	230 (218–239)
Spring 2021	META (42)	2 (4.8)	0 (0)	232 (219–241)
	NO META (40)	5 (12.5)	0 (0)	229 (219–244)
	All (82)	7 (8.5)^c^	0 (0)^e^	230 (219–242)
Fall 2021	META (42)	19 (45.2)	5 (11.9)	234 (227–249)
	NO META (42)	17 (40.5)	8 (19.0)	238 (232–246)
	All (84)	36 (42.9)^d^	13 (15.5)^f^	237 (229–248)
Overall	META (167)	49 (29.3)	8 (4.8)	231 (220–242)
	NO META (164)	33 (20.1)	9 (5.5)	233 (220–244)
	All (331)	72 (21.8)	17 (5.1)	232 (220–242)

*Group numbers are after removals. Values with different superscripts in the same column of the ‘META’ or ‘NO META’ rows represent a statistical difference between TxGroups within Trial (ab, *χ*^2^, *p* < 0.05). Values with different superscripts in the same column of the ‘All’ rows represent a statistically significant difference among Trials (cd or ef, Pairwise Fisher’s Exact test with Benjamini-Hochberg adjustment, *p* < 0.05). Percentages represent percent of animals. *MH*, *Mannheimia haemolytica*; MDR, multi-drug resistant; SD, standard deviation; META, tulathromycin metaphylaxis; NO META, no tulathromycin metaphylaxis; TxGroup, treatment group.

#### 3.4.1 Antimicrobial susceptibility testing

Minimum inhibitory concentrations and interpretations of individual antimicrobials tested are listed for each isolate in Table S4. Tetracycline was the antimicrobial most commonly found as not susceptible (31% of arrival isolates).

#### 3.4.2 Antimicrobial resistance genes

**Figure 3:**
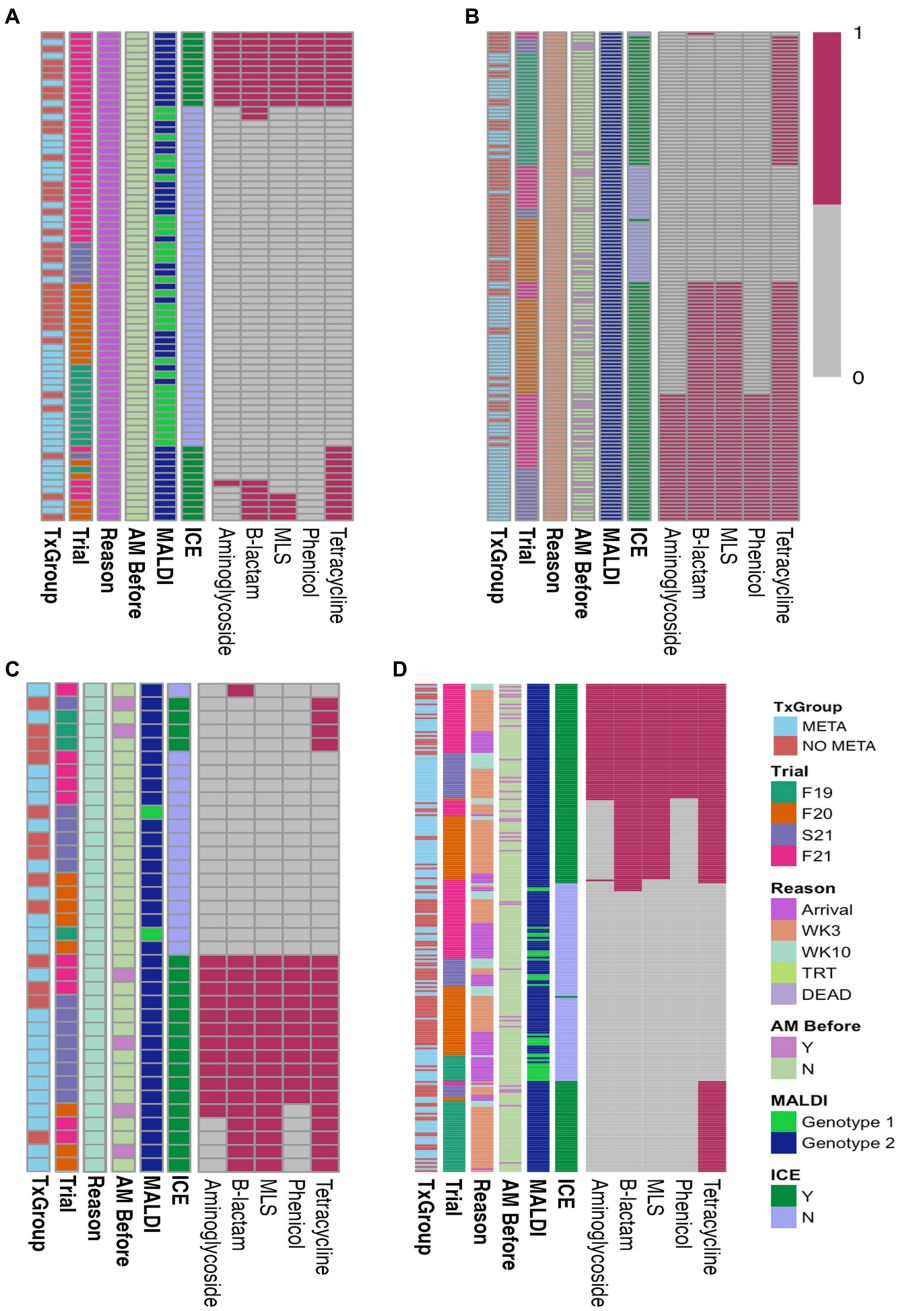
Heatmaps of ARGs in isolated *MH*. **(A)** Arrival isolates, **(B)** WK3 isolates, **(C)** WK10 isolates, and **(D)** all isolates combined. AM Before, treated with antimicrobial before isolation; MALDI, MALDI biomarker genotype; F19, Fall 2019; F20, Fall 2020; S21, Spring 2021; F21, Fall 2021; META, metaphylaxis; NO META, no metaphylaxis; ICE, presence of ICE; Y, Yes; N, No; WK3, week 3; WK10, week 10; ICE, integrative conjugative element; MLS, macrolides-lincosamides-streptogrammin. Key: 1 = present, 0 = absent.

Of the 72 arrival isolates, 48 (67%) had no antimicrobial resistance genes found (Figure 3A; Table S3), and the most commonly identified resistance gene was *tetH*, which was identified in 22 (31%) arrival isolates.

Overall, there was no differences in the likelihood of isolation of *MH* or MDR *MH* at arrival between TxGroup (Figure 2A, Table 1, *χ*^2^, *p* > 0.05); however, in Fall 2019, there were significantly greater numbers of *MH* isolated on arrival from calves in the META TxGroup compared to the NO META (Table 1, *χ*^2^, *p* = 0.016). There was also significant variation in the number of MDR *MH* isolates recovered at arrival among the Trials with Spring 2021 having significantly more than Fall 2019 and Fall 2021 (Table 1, Pairwise Fisher’s Exact test, *p* < 0.05).

### 3.5 Isolation of MDR *MH*

Heatmaps of AST results (Figure 2) and presence of ARGs at the class level (Figure 3) show the distribution of antimicrobial resistant *MH* isolates between TxGroups at arrival. The majority of MDR isolates recovered at 3 weeks after arrival (Figures 2B, 3B) contained ICE genes and were from animals that received metaphylaxis. This was also true at week 10 (Figures 2C, 3C). Most ICE negative isolates across all time points did not contain any ARGs (Figure 3D).

**Figure 4:**
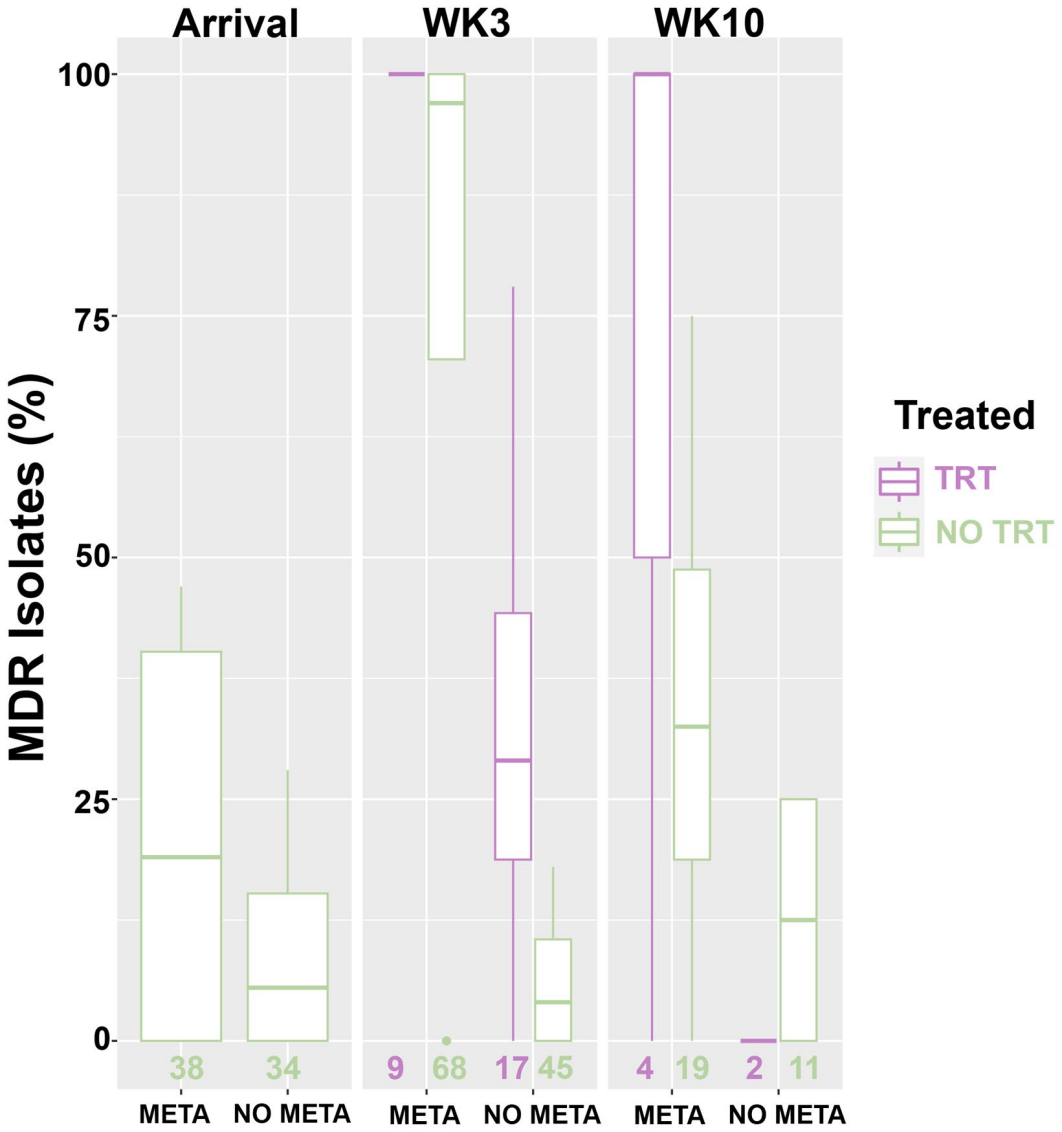
Box and whisker plot of isolation rate of MDR *MH*. *MH, Mannheimia haemolytica*; TxGroup, treatment group; MDR, multidrug resistant; META, tulathromycin metaphylaxis; NO META, no tulathromycin metaphylaxis; TRT, treated; NO TRT, not treated; n, total number of *MH* isolates.

There was an increase in isolation of *MH* (43% of animals) and MDR *MH* (50% of *MH* isolates) at week 3 compared to arrival (22% *MH*, 5.1% MDR *MH*; Table 2, McNemar’s *χ*^2^ test, *p* < 0.0001; Figures 2A,B,D, 4). There were significantly different numbers of *MH* and MDR *MH* isolated at arrival, isolated at week 3, and isolated at week 10 across Trials (Table 2, Pairwise Fisher’s Exact Test, *p* < 0.05). There were also increased numbers of isolates with resistance genes to multiple antimicrobial classes at week 3 compared to arrival (Table 3, McNemar’s *χ*^2^ test, *p* < 0.0001; Figure 3B). Isolation of *MH* and MDR *MH* at arrival, 3, and 10 weeks in animals that were treated for BRD are shown in Table S8.

**Table 2:** *Mannheimia haemolytica* isolation at arrival, week 3, and week 10 across 4 separate trials.

Trial	TxGroup	N_Arr_	*MH*_Arr_ n (%)	MDR_Arr_ n (%)	N_WK3_	*MH*_WK3_ n (%)**	MDR_WK3_ n (%)**	N_WK10_	*MH*_WK10_ n (%)	MDR_WK10_ n (%)
Fall 2019	META	41	10 (24)^a^	0 (0)	41	20 (49)	0 (0)	41	2 (5)	0 (0)
	NO META	41	2 (4.9)^b^	0 (0)	41	12 (29)	0 (0)	41	2 (5)	0 (0)
	All	82	12 (15)^c^	0 (0)^e^	82	32 (39)^gh^	0 (0)^m^	82	4 (5)^o^	0 (0)^q^
Fall 2020	META	42	8 (19)	3 (7.1)	41	24 (58)	24 (59)^i^	40	6 (15)	3 (8)
	NO META	41	9 (22)	1 (2.4)	39	20 (51)	4 (10)^j^	39	2 (5)	0 (0)
	All	83	17 (20)^c^	4 (4.8)^ef^	80	44 (55)^g^	28 (35)^n^	79	8 (10)^op^	3 (4)^qr^
Spring 2021	META	42	2 (4.8)	0 (0)	40	15 (38)	15 (38)^k^	38	9 (24)	7 (18)
	NO META	40	5 (12)	0 (0)	36	7 (19)	1 (3)^l^	33	5 (15)	1 (3)
	All	82	7 (8.5)^c^	0 (0)^e^	76	22 (29)^h^	16 (21)^n^	71	14 (20)^p^	8 (11)^r^
Fall 2021	META	42	19 (45)	5 (12)	42	18 (43)	17 (40)	42	6 (14)	2 (5)
	NO META	42	17 (40)	8 (19)	41	22 (54)	8 (20)	40	4 (10)	1 (3)
	All	84	36 (43)^d^	13 (16)^f^	83	40 (48)^g^	25 (30)^n^	82	10 (12)^op^	3 (4)^qr^
Overall	META	167	39 (29)	8 (4.8)	164	77 (47)	56 (34)	161	23 (14)	12 (7)
	NO META	164	33 (20)	9 (5.5)	157	61 (39)	13 (8)	153	13 (8)	2 (1)
	All	331	72 (22)	17 (5.1)	321	138 (43)	69 (21)*	321	36 (11)	14 (4)*

An asterisk (*) in the ‘Overall’ row represents that there was an overall statistical difference between META and NO META TxGroups (*χ*^2^ test, *p* < 0.05). A double asterisk (**) in the header indicates there was a statistically significant difference between a measurement compared to arrival (McNemar’s *χ*^2^ test, *p* < 0.0001). Values with different superscripts in the same column of the ‘META’ or ‘NO META’ rows represent a statistical difference between TxGroups within Trial (ab, *χ*^2^, *p* < 0.05). Values with different superscripts in the same column of the ‘All’ rows represent a statistically significant difference among Trials (cd, ef, gh, mn, op, or qr Pairwise Fisher’s Exact test with Benjamini-Hochberg adjustment, *p* < 0.05). *MH*, *Mannheimia haemolytica*; MDR, multi-drug resistant; Arr, arrival; WK3, week 3; WK10, week 10; N, number of animals sampled; META, tulathromycin metaphylaxis; NO META, no tulathromycin metaphylaxis; TxGroup, treatment group. Percentage of *MH* isolated is based on total animals sampled. Percentage of MDR is based on total animals sampled.

**Table 3:** Resistance genes identified in *MH* isolated from study animals.

TxGroup (Isolates)	Isolates with ARGs (Multiple ARGs)	Amin (a_3”,_ a_6_)	B-lac (b_O_, b_R_)	MLS (m_e_, m_m_)	Phen	Tet (t_H_, t_R_)	Total ARGs
META (139 total)	102 (75)	43 (1, 42)	77 (73, 34)	73 (73, 73)	41	100 (100, 51)	491
Arrival (39)	13 (8)	5 (0, 5)	9 (6, 9)	6 (6, 6)	4	12 (12, 4)	52
WK3 (77)	75 (55*)	29 (0, 29)	55 (55, 23)	55 (55, 55)	29	75 (75, 35)	360
WK10 (23)	14 (12)	9 (1, 8)	13 (12, 3)	12 (12, 12)	8	13 (13, 10)	79
NO META (108 total)	59 (26)	17 (0, 17)	28 (25, 25)	26 (25, 26)	17	47 (47, 13)	196
Arrival (33)	11 (9)	7 (0, 7)	10 (9, 9)	9 (9, 9)	7	10 (10, 4)	65
WK3 (62)	31 (13)	7 (0, 7)	14 (12, 13)	13 (12, 13)	7	30 (30, 6)	100
WK10 (13)	7 (4)	3 (0, 3)	4 (4, 3)	4 (4, 4)	3	7 (7, 3)	31
Grand total (246)	151 (101)	60 (1, 59)	105 (98, 59)	99 (98, 99)	58	147 (147, 62)	687

*Significant difference in isolation of MDR *MH* compared to previous time point (McNemar’s *χ*^2^ test, *p* < 0.0001). TxGroup, treatment group; META, tulathromycin metaphylaxis; NO META, no tulathromycin metaphylaxis; ARG, antimicrobial resistance gene; WK3, week 3; WK10, week 10; Amin, aminoglycoside; B-lac, beta-lactams; MLS, macrolide-lincosamide-streptogramins; Phen, phenicols; Tet, tetracyclines; a_3”,_*aph(3″)*; a_6’_, *aph(6′)*; b_o_, *bla*_OXA_; b_R_, *bla*_ROB_; m_e_, *erm(42)*; m_m_, *msr(E)*; t_H_, *tetH;*t_R_, *tetR*.

**Figure 5:**
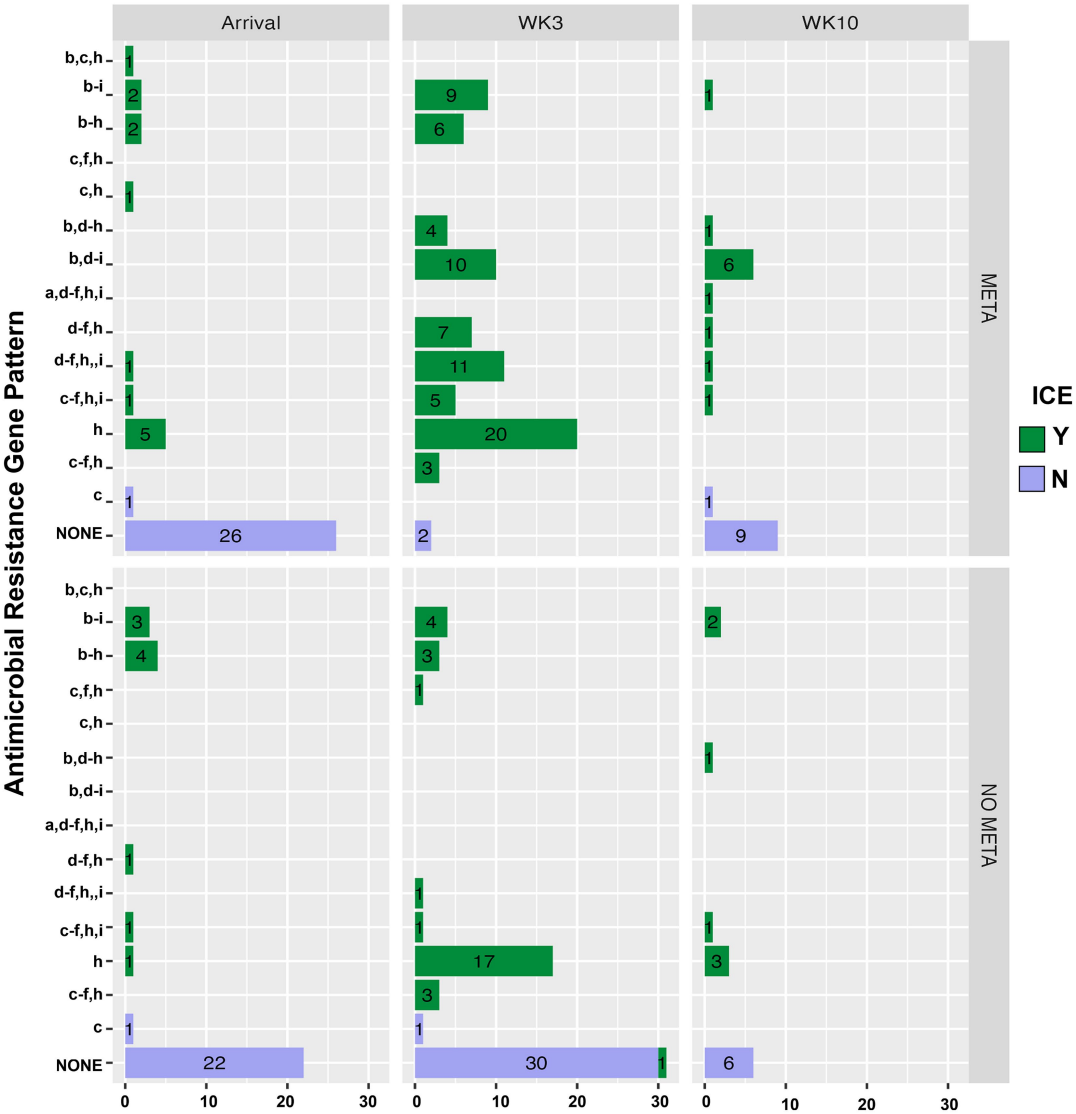
Antimicrobial resistance gene pattern of isolated *MH*, faceted by TxGroup and sampling time. Numbers indicate total number of isolates with that resistance pattern. (a) aph(3′), (b) aph(6′), (c) blaROB, (d) blaOXA, (e) erm(42), (f) msr(E), (g) floR, (h) tetH, and (i) tetR. *MH, Mannheimia haemolytica*; TxGroup, treatment group; MDR, multidrug resistant; META, tulathromycin metaphylaxis; NO META, no tulathromycin metaphylaxis; TRT, treated; NO TRT, not treated.

Figure 5 is a bar plot of the resistance gene patterns colored by ICE presence and separated by TxGroup and sampling timepoint. Isolation of ICE-positive *MH* increased overall and within-TxGroups from arrival to week 3 (Figure 5). There were 15 unique antimicrobial resistance gene patterns found in all isolates (Table S9; Figure 5). The most common ARG pattern found in ICE-positive *MH* isolated at any time (*n* = 148) was *tetH* alone (*n* = 46, META = 25, NO META = 21, Figure 5). Of ICE-negative isolates (*n* = 99), ninety-five (META = 37, NO META = 58) had no resistance genes found, whereas the other 4 contained *bla*_ROB_. Only 1 ICE-positive isolate (NO META, WK3) contained no resistance genes. Also of interest, only one isolate harbored *msr(E)* without *mph(42)* (NO META, WK10); in all other isolates *mph(42)* and *msr(E)* were found together. Similarly, no isolates were found with *tetR* without *tetH*. The most common individual antimicrobial resistance gene at all timepoints was *tetH*. Indeed, in isolates containing resistance genes (*n* = 151), only 4 did not contain *tetH* (Figure 5; Table S9).

### 3.6 Factors associated with isolation of *MH* and MDR *MH*

#### 3.6.1 Samples collected 3-weeks after arrival

Factors significantly associated with odds of isolation of *MH*, MDR *MH*, or ICE-positive *MH* at 3 weeks are shown in Table S10 and Table 4. Variables included in modeling of *MH* isolation in samples collected after 3 weeks were TxGroup, Fever at Arrival, and Difference from median weight at arrival (Table S10, *p <* 0.2). For modeling risk of MDR *MH* Isolation at 3 weeks, TxGroup, Fever at Arrival, and BRD morbidity were eligible for inclusion into the final multivariable model. TxGroup and Fever at Arrival were screened for inclusion in final models regarding risk of ICE-positive *MH* at 3 weeks.

**Table 4:** Final models for *MH* isolation outcomes at 3 weeks.

Outcome	Variable	Value	OR	95% CI	*p*-value
*MH* isolation	TxGroup	META	1.41	0.90–2.22	0.13
		NO META	Ref	Ref	Ref
MDR *MH* isolation	TxGroup	META	13.08	5.54–30.88	<0.0001
		NO META	Ref	Ref	Ref
	BRD Treatment x FeverAR	Interaction	–	–	0.02
		N x N	Ref	Ref	Ref^ab^
		Y x Y	9.70	1.63–57.78	0.061^ac^
		N x Y	0.18	0.05–0.65	0.009^b^
		Y x N	3.83	1.39–10.58	0.01^c^
Isolation of *MH* containing ICE	TxGroup	META	3.43	2.08–5.66	<0.0001
		NO META	Ref	Ref	Ref

BRD treatment (Yes or No) indicates whether an animal received antimicrobials for BRD treatment before 3 week sampling. Weight at arrival input is difference from median weight (232 kg). TxGroup, treatment group; BRD, bovine respiratory disease; MDR, multidrug resistant; *MH*, *Mannheimia haemolytica*; Ref, reference; OR, Odds Ratio; CI, confidence interval; META, tulathromycin metaphylaxis; NO META, no tulathromycin metaphylaxis. Values in the same column with different superscripts indicate statistically significant difference (Pairwise Comparison of LS Means Differences, *p* < 0.05).

There was no significant association between any variable tested on isolation of *MH* at 3 weeks [Table 4, META, OR (95%CI) = 1.41 (0.9–2.22), *p* = 0.13]. Importantly, animals that received metaphylaxis had over 13 times higher odds of isolation of MDR *MH* compared to those that did not [Table 4, META, OR (95 %CI) = 13.08 (5.54–30.9), *p* < 0.0001]. There was a significant interaction between BRD treatment and Fever at arrival (Table 4, *p* = 0.02). Specifically, in animals that were febrile at arrival, there was higher odds of isolation of MDR *MH* from animals that required BRD treatment from those that did not (Table 4); a similar association was seen in afebrile animals. Additionally, there were also increased odds of isolation of *MH* containing ICE at 3 weeks from animals that had received metaphylaxis [Table 4, META, OR (95 %CI) = 3.43 (2.08–5.66), *p* < 0.0001].

#### 3.6.2 Samples collected 10 weeks after arrival

Factors that were significantly associated with the odds of isolation of *MH*, MDR *MH*, or ICE-positive *MH* at 10 weeks after arrival are shown in Table S11 and Table 5. Variables screened for inclusion in modeling of week 10 *MH* isolation were TxGroup (*p* = 0.14), Fever at Arrival (*p* = 0.14), isolation of *MH* at arrival (*p =* 0.14) and at week 3 (Table S11, *p* = 0.08). For modeling risk of MDR *MH* Isolation at 10 weeks, TxGroup and isolation of MDR *MH* at 3 weeks were eligible for inclusion into the final multivariable model (Table S11). Treatment group and isolation of *MH* at arrival were eligible for modeling isolation of ICE-positive *MH* (Table S11, *p* = 0.21 and *p =* 0.14, respectively).

**Table 5:** Final models for *MH* isolation outcomes at 10 weeks.

Outcome	Variable	Value	OR	95% CI	*p*-value
*MH* isolation	TxGroup	META	1.72	0.84–3.55	0.14
		NO META	Ref	Ref	Ref
MDR *MH* isolation	TxGroup	META	5.92	1.34–26.14	0.019
		NO META	Ref	Ref	Ref
Isolation of *MH* containing ICE	TxGroup	META	1.88	0.69–5.12	0.21
		NO META	Ref	Ref	Ref

BRD treatment (Yes or No) indicates whether an animal received antimicrobials for BRD treatment before 10 week sampling. TxGroup, treatment group; *MH*, *Mannheimia haemolytica*; BRD, bovine respiratory disease; MDR, multidrug resistant; META, tulathromycin metaphylaxis; NO META, no tulathromycin metaphylaxis; Ref, reference; OR, Odds Ratio; CI, confidence interval; ICE, integrative conjugative element.

There was no significant association between any variable tested and odds of isolation of *MH* or ICE-positive *MH* at 10 weeks (Table 5). Importantly, animals that received metaphylaxis had about 6-times higher odds of isolation of MDR *MH* at 10 weeks compared to those that did not (Table 5, META, OR (95 %CI) = 5.9 (1.34–26.14), *p* = 0.02).

### 3.7 Animal health: weight gain, morbidity, and mortality

#### 3.7.1 Health and production from arrival to 3 weeks

Average daily gain, crude morbidity, BRD-specific morbidity, and crude mortality results are provided in Table 6. Overall, animals that received metaphylaxis [ADG (95% CI) = 1.02 (0.43–1.49) kg/day], gained more weight compared to those that did not [0.70 (0.16–1.18) kg/day] over the first 3 weeks after arrival (Table 6, Wilcoxon Sum Rank Test, *p* = 0.0002). This increase in weight gain was found in all Trials except for Spring of 2021 (Table 6; Wilcoxon Sum Rank Test; Fall 2019, *p =* 0.003; Fall 2020, *p* = 0.04; Spring 2021, *p* = 0.66; Fall 2021, *p* = 0.01). There were fewer animals treated in META, 15% (15/167), compared to NO META, 29% (29/164) (Table 6, *χ*^2^ test, *p* = 0.0007). This was also the case for BRD treatment (META, 12% (20/167); NO META, 28% (46/164); Table 6, *χ*^2^ test, *p* = 0.0007). When separated by Trial, there were no significant differences in crude or BRD-specific morbidity in Fall 2019 or Fall 2020 (Table 6, *χ*^2^ test, *p* > 0.05). There was not a statistically significant difference in mortality between TxGroup or among Trials (Table 6, *χ*^2^ test and Fisher’s Exact Test, *p* > 0.05).

**Table 6:** Animal health from arrival to 3 weeks.

Trial	TxGroup	n	ADG (95% CI) (kg)	Animals treated n (%)^#^	BRD morbidity n (%)^#^	Crude mortality n (%)
Fall 2019	META	41	1.48 (1.20–1.77)^d^	11 (27)	9 (22)	0 (0)
	NO META	41	1.00 (0.66–1.30)^e^	9 (22)	7 (17)	0 (0)
	All	82	1.21 (0.83–1.65)^j^	20 (24)	16 (20)	0 (0)
Fall 2020	META	42	0.54 (0.22–1.08)^f^	6 (19)	6 (14)	1 (2)
	NO META	41	0.22 (−0.22–0.70)^g^	11 (27)	10 (24)	2 (5)
	All	83	0.43 (−0.11–0.97)^k^	17 (20)	16 (19)	3 (4)
Spring 2021	META	42	0.88 (0.34–1.27)	5 (10)^m^	4 (10)^q^	2 (5)
	NO META	40	0.69 (0.22–1.37)	18 (42)^n^	17 (44)^r^	4 (10)
	All	82	0.83 (0.26–1.34)^l^	23 (26)	21 (26)	6 (7)
Fall 2021	META	42	1.02 (0.57–1.49)^h^	2 (2)^o^	1 (2)^s^	0 (0)
	NO META	42	0.78 (−0.02–1.13)^i^	12 (29)^p^	12 (29)^t^	1 (2)
	All	84	0.91 (0.34–1.23)^l^	14 (16)	13 (16)	1 (1)
Overall	META	167	1.02 (0.43–1.49)	24 (15)	20 (12)	3 (2)
	NO META	164	0.70 (0.16–1.18)	50 (29)	46 (28)	7 (4)
	All	331	0.89 (0.30–1.34)*^a^	74 (22)*^b^	66 (20)*^c^	10 (3)

An asterisk (*) in the ‘Overall’ row represents that there was an overall statistical difference between META and NO META TxGroups (a, Wilcoxon Sum Rank test, *p* = 0.0002; b, *χ*^2^ test, *p* = 0.0007; c, *χ*^2^ test, *p =* 0.004). Values with different superscripts in the same column of the ‘META’ or ‘NO META’ rows represent a statistical difference between TxGroups within Trial (d-i, Wilcoxon Sum Rank, *p* ≤ 0.05; m-p and q-t, *χ*^2^ test, *p* ≤ 0.05). Values with different superscripts in the same column of the ‘All’ rows represent a statistically significant difference among Trials (j-l, Kruskal Wallis Analysis of Variance on Ranks with *post-hoc* Dunn Test and Benjamini Hochberg Correction, *p* ≤ 0.05). Percentages represent percent of animals. ^#^There was a significant difference between animals treated with antimicrobials and animals treated for BRD (McNemar’s *χ*^2^ Test, *p* = 0.01). TxGroup, treatment group; *MH*, *Mannheimia haemolytica*; MDR, multi-drug resistant; CI, confidence interval; ADG, average daily gain; BRD, bovine respiratory disease; META, tulathromycin metaphylaxis; NO META, no tulathromycin metaphylaxis.

##### 3.7.1.1 Factors associated with animal health at 3 weeks

Mixed effect logistic models for BRD morbidity and crude mortality and mixed effect linear models for ADG over 3 weeks after arrival are shown in Table S12 and Table 7. Variables screened for modeling of BRD morbidity included TxGroup (Table S12, *p =* 0.004) and arrival weight (*p* = 0.011). Variables eligible for inclusion of the final model of mortality were TxGroup (*p* = 0.19), isolation of *MH* containing ICE at arrival (*p* = 0.046) or being treated for BRD within 3 weeks after arrival (*p* < 0.0001). TxGroup (*p* < 0.0001), fever at arrival (*p* = 0.007), weight at arrival (*p =* 0.16), and being treated for BRD (*p* < 0.0001), were evaluated for inclusion into the final linear mixed effects model of ADG.

**Table 7:** Final models for health outcomes from arrival through 3 weeks.

Outcome	Variable	Value		OR/Est	95% CI/SE	*p*-value
BRD Morbidity	TxGroup	META		Ref	Ref	Ref
		NO META		3.07	1.70–5.52	0.0002
	Weight at arrival	232 (kg)		Ref	Ref	Ref
		Difference (kg)		0.98	0.96–0.99	0.006
Crude Mortality	TxGroup	META		Ref	Ref	Ref
		NO META		1.19	0.27–5.20	0.82
	BRD Treatment	Yes		16.68	3.33–83.45	0.0006
		No		Ref	Ref	Ref
Average daily gain	TxGroup	META		0.23	0.07	0.002
		NO META		Ref	Ref	Ref
	BRD Treatment x FeverAR	BRD	Fever	−0.71	0.27	0.01
		Y	Y	Ref	Ref	Ref^a^
		N	N	1.73	0.24	0.008^b^
		Y	N	0.91	0.25	0.0003^c^
		N	Y	1.54	0.26	<0.0001^b^

BRD Morbidity is the number of animals treated at least once for BRD over first 3 weeks of the study period. Crude Mortality includes all animals who died over the first 3 weeks after arrival. ^#^TxGroup (META or NO META) was included in all multivariable models, regardless of *p*-value. Weight at arrival input is difference in kg from median weight (232 kg). TxGroup, treatment group; BRD, bovine respiratory disease; ADG, average daily gain (kg/day); *MH, Mannheimia haemolytica*; MDR, multidrug resistant; FeverAR, fever at arrival (rectal temperature > 40°C); Ref, reference; OR, Odds Ratio; CI, confidence interval; SE, standard error; Est, Restricted Maximum Likelihood estimate; META, tulathromycin metaphylaxis; NO META, no tulathromycin metaphylaxis. Values in the same column with different superscripts indicate statistically significant difference (Pairwise Comparison of LS Means Differences, *p* < 0.05).

Animals that did not receive metaphylaxis (Table 7, NO META, OR (95% CI) = 3.07 (1.70–5.52), *p =* 0.002) had 3 times greater odds of being treated for BRD in the first 3 weeks of the study period, and for every kg increase in arrival weight compared to median arrival weight (232 kg, 95% CI = 220–242 kg), there was decrease in odds of being treated for BRD (Table 7, OR (95% CI) = 0.98 (0.96–0.99), *p =* 0.006).

Metaphylaxis was not significantly associated with odds of dying during the first 3 weeks after arrival [Table 7, NO META, OR (95% CI) = 1.19 (0.27–5.2), *p* = 0.82]; however, animals that were treated for BRD had approximately 16 times higher odds of dying compared to those that were not treated for BRD [Table 7, No BRD, OR (95% CI) = 16.68 (3.33–83.45), *p* = 0.006].

Finally, animals that received metaphylaxis gained approximately 0.2 kg/day more than animals that did not [Table 7, META, Est (SE), 0.23 kg/day (0.07), *p* = 0.002]. The interaction between treatment for BRD and fever at arrival was significantly associated with average daily gain, with animals that were febrile at arrival and being treated for BRD having lower average daily gain compared to all other combinations (Table 7). Further, in afebrile animals, BRD treatment was significantly associated with decreased weight gain within 3 weeks after arrival.

#### 3.7.2 Health and production from arrival to 10 weeks

Average daily gain, overall and BRD morbidity, and mortality over 10 weeks are listed in Table 8. Overall, animals that received metaphylaxis, ADG (95% CI) = 0.80 (0.51–0.97) kg/day, gained more weight compared to those that did not, 0.64 (0.44–0.89) kg/day, over the entirety of the study period (Table 8, Wilcoxon Sum Rank Test, *p* = 0.002). This increase in ADG was found in all Trials except for Spring of 2021 (Table 8; Wilcoxon Sum Rank Test; Fall 2019, *p =* 0.003; Fall 2020, *p* = 0.04; Spring 2021, *p* = 0.66; Fall 2021, *p* = 0.01). There were fewer animals treated in the META TxGroup, 19% (31/167), compared to the NO META TxGroup, 34% (55/164) (Table 8, *χ*^2^ test, *p* = 0.003). This was also the case for BRD treatment (META, 14% (24/167); NO META, 30% (50/164); Table 8, *χ*^2^ test, *p* = 0.0007). When separated by Trial, there was no significant difference in BRD morbidity in Fall 2019 or Fall 2020 (Table 8, *χ*^2^ test, *P*_Fall2019_ = 1.0, *P_Fall2020_* = 0.55). There was no statistically significant difference in mortality between META and NO META TxGroups or among Trials (Table 8, *χ*^2^ test and Fisher’s Exact Test, *p* > 0.05).

**Table 8:** Week 10 health data.

Trial	TxGroup	*n*	Median-ADG (95% CI) (kg)	Animals treated n (%)^#^	BRD Morbidity n (%)^#^	Mortality n (%)
Fall 2019	META	41	0.96 (0.87–1.17)^d^	11 (27)	9 (22)	0 (0)
	NO META	41	0.88 (0.71–0.95)^e^	11 (27)	9 (22)	0 (0)
	All	82	0.92 (0.79–1.08)^j^	22 (27)	18 (22)	0 (0)
Fall 2020	META	42	0.82 (0.64–0.92)^f^	9 (21)	7 (17)	2 (5)
	NO META	41	0.65 (0.42–0.86)^g^	12 (29)	10 (24)	2 (5)
	All	84	0.78 (0.49–0.91)^k^	21 (25)	17 (20)	4 (5)
Spring 2021	META	42	0.76 (0.61–0.97)	5 (12)^m^	4 (10)^o^	4 (10)
	NO META	40	0.73 (0.52–0.88)	18 (45)^n^	17 (43)^p^	7 (18)
	All	82	0.76 (0.53–0.94)^k^	23 (28)	21 (26)	11 (13)
Fall 2021	META	42	0.45 (0.33–0.71)^h^	6 (14)	4 (10)^q^	0 (0)
	NO META	42	0.38 (0.19–0.57)^i^	14 (33)	14 (33)^r^	2 (5)
	All	84	0.44 (0.25–0.62)^l^	20 (24)	18 (21)	2 (2)
Overall	META	167	0.80 (0.51–0.97)	31 (19)	24 (14)	6 (4)
	NO META	164	0.64 (0.44–0.89)	55 (34)	50 (30)	11 (7)
	All	331	0.74 (0.45–0.92)*^a^	86 (26)*^b^	74 (22)*^c^	17 (5)

An asterisk (*) in the ‘Overall’ row represents that there was an overall statistical difference between META and NO META TxGroups (a, Wilcoxon Sum Rank test, *p* = 0.002; b, *χ*^2^ test, *p =* 0.002; c, *χ*^2^ test, *p =* 0.0007). Values with different superscripts in the same column of the ‘META’ or ‘NO META’ rows represent a statistical difference between TxGroups within Trial (d-i, Wilcoxon Sum Rank, *p ≤* 0.05; mn, op, and qr, *χ*^2^ test, *p ≤* 0.05). Values with different superscripts in the same column of the ‘All’ rows represent a statistically significant difference among Trials (j-l, Kruskal Wallis Analysis of Variance on Ranks with *post-hoc* Dunn Test and Benjamini Hochberg Correction, *p ≤* 0.05). ^#^There was a significant difference between animals treated with antimicrobials and animals treated for BRD (McNemar’s *χ*^2^ Test, *p* = 0.001). Percentages represent percent of animals. TxGroup, treatment group; *MH*, *Mannheimia haemolytica*; MDR, multi-drug resistant; CI, confidence interval; ADG, average daily gain; BRD, bovine respiratory disease; META, tulathromycin metaphylaxis; NO META, no tulathromycin metaphylaxis.

##### 3.7.2.1 Factors associated with animal health at 10 weeks

Mixed-effects logistic regression models for 10-week BRD morbidity and mortality and mixed-effects linear regression models for ADG are shown in Table S13 and Table 9. Variables screened for modeling BRD morbidity included TxGroup (Table S13, *p =* 0.0006), isolation of MDR *MH* at WK3 (*p =* 0.18) and arrival weight (*p* = 0.02). Variables eligible for inclusion in the final model of mortality were TxGroup (*p* = 0.17), isolation of *MH* at arrival (*p* < 0.0001), isolation of MDR *MH* at arrival and WK3 (*p* = 0.046, *p* = 0.2), isolation of *MH* containing ICE at arrival and WK3 (*p* = 0.01, *p* = 0.12), isolation of genotype 2 *MH* at arrival (*p* = 0.04), and being treated for BRD during the first 10-weeks after arrival (*p* < 0.0001). TxGroup (*p < 0.01*), fever at arrival (*p* = 0.11), weight at arrival (*p =* 0.14), and being treated for BRD (*p* < 0.0001) were tested for inclusion in the final linear mixed model of ADG over 10 weeks.

**Table 9:** Final models for health outcomes from arrival through 10 weeks.

Outcome	Variable	Value	OR/Est	95% CI/SE	*p*-value
BRD Morbidity	TxGroup	META	Ref	Ref	Ref
		NO META	2.76	1.59–4.80	0.0002
	Weight at arrival	232 (kg)	Ref	Ref	Ref
		Difference (kg)	0.98	0.96–0.99	0.012
Crude Mortality	TxGroup	META	Ref	Ref	Ref
		NO META	1.24	0.38–4.08	0.72
	BRD treatment	Yes	23.62	6.08–91.78	<0.0001
		No	Ref	Ref	Ref
Average daily gain	TxGroup	META	0.21	0.08	0.006
		NO META	Ref	Ref	Ref

BRD Morbidity is the number of animals treated at least once for BRD over first 10 weeks of study period. Mortality includes all animals who died through 10 weeks after arrival. Weight at arrival input is difference from median weight (232 kg). TxGroup, treatment group; BRD, bovine respiratory disease; ADG, average daily gain (kg/day); *MH, Mannheimia haemolytica*; MDR, multidrug resistant; Ref, reference; OR, Odds Ratio; CI, confidence interval; SE, standard error; Est, Restricted Maximum Likelihood estimate; META, tulathromycin metaphylaxis; NO META, no tulathromycin metaphylaxis.

Animals that did not receive metaphylaxis [Table 9, NO META, OR (95% CI) = 2.76 (1.59–4.80), *p =* 0.0002] had around 2.5 times greater odds of being treated for BRD during the entire study period, and for every kg increase in arrival weight compared to median arrival weight (232 kg, 95% CI = 220–242 kg), there was decrease in odds of being treated for BRD [Table 9, OR (95% CI) = 0.98 (0.96–0.99), *p =* 0.012].

Metaphylaxis was not significantly associated with odds of dying [Table 9, META, OR (95% CI) = 1.24 (0.38–4.08), *p* = 0.72]; however, animals that were treated for BRD had over 23 times higher odds of dying compared to those that were not treated for BRD [Table 9, BRD, OR (95% CI) = 23.62 (6.08–91.78), *p* < 0.0001].

Finally, animals that received metaphylaxis gained approximately 0.2 kg/day more than animals that did not [Table 9, META, Est (SE), 0.23 (0.07), *p* = 0.002].

### 3.8 Phylogenic analysis of *MH* isolates

**Figure 6:**
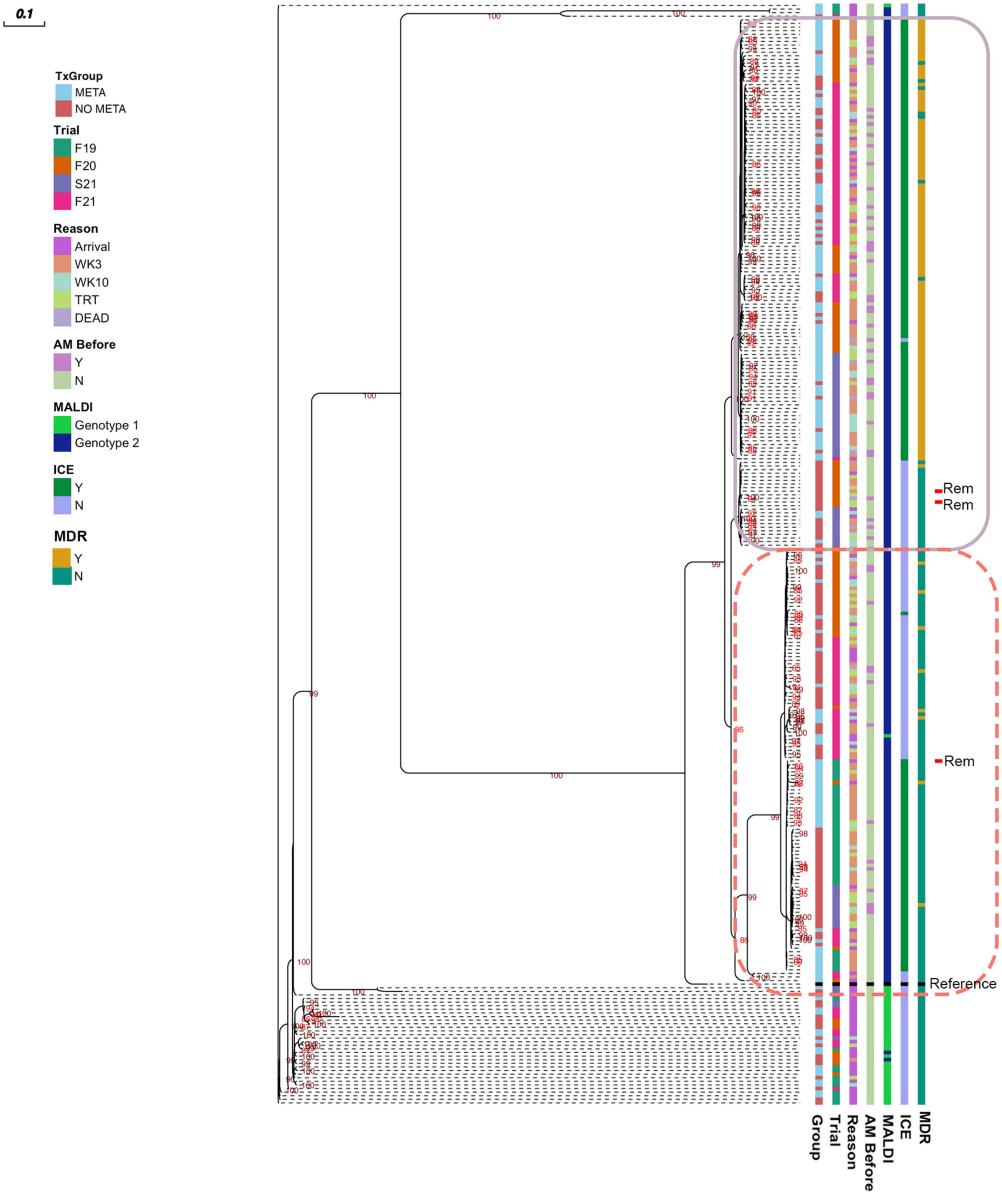
Maximum likelihood tree of pangenome of all *MH* isolated. Numbers indicate ultrafast bootstrap values generated from 1000 replicates; values less than 85 are not shown. Outlines indicate clustered isolates. *MH*, *Mannheimia haemolytica*; TxGroup, treatment group; AM Before, treated with antimicrobial before isolation; MALDI, MALDI biomarker genotype; F19, Fall 2019; F20, Fall 2020; S21, Spring 2021; F21, Fall 2021; META, tulathromycin metaphylaxis; NO META, no tulathromycin metaphylaxis; ICE, integrative conjugative element; Y, Yes; N, No; WK3, week 3; WK10, week 10; Rem, isolate from animal removed from study; MDR, Multidrug Resistant. Reference- *Mannheimia haemolytica* USDA-ARS-USMARC-191 (GenBank: CP023044.1).

A midpoint rooted, Maximum Likelihood phylogenetic tree is shown in Figure 6. There are 2 clusters of genomes with little genetic variation (circled with solid and dashed lines), and one cluster that has higher genetic variability. Sequences clustered well by MALDI genotype, and *MH* isolated at arrival had higher genetic variability than later in the feeding period (Figure 6). The majority of MDR *MH* are within one cluster (solid line, Figure 6).

## 4 Discussion

### 4.1 Tulathromycin metaphylaxis is associated with increased isolation of MDR *MH*

With the present study, we demonstrated that isolation of MDR *MH* at 3 and 10 weeks was much more likely in calves that received tulathromycin metaphylaxis at arrival than those that did not (Tables 4, 5 and Figures 2–5), despite a lack of overall difference in the likelihood of isolation of *MH* (susceptible and MDR). This increase in MDR *MH* isolation is similar to findings previously described (18, 19). However, in both prior studies, all animals received macrolides at arrival, with no inclusion of negative-control animals, so the effect of metaphylaxis could not be separated from other factors. In a longitudinal study following calves from ‘branding’ to ‘weaning’ to ‘reprocessing’ at feedlots in Western Canada, Nobrega et al. (20) examined risk factors associated with AMR in BRD pathogens. In this study, there was reduced susceptibility to tulathromycin in *MH* at reprocessing from animals that had received an injectable macrolide at or around the time of introduction into the feedlot; however, isolation of *MH* in that study was relatively rare (<5% of animals sampled across all time points). The bacterial species most commonly cultured from NPS in this study was *Pasteurella multocida*, another member of the family Pasteurellaceae commonly associated with BRD, which has also demonstrated MDR associated with ICE. One additional major difference is the timing of second sampling between the two studies. The ‘reprocessing’ that was used to evaluate the effect of metaphylaxis given near the time of arrival at the feedlot occurred at an average of 142 days on feed. In the present study, peak isolation of *MH* was at 3 weeks after arrival, consistent with previous results for stocker calves in Mississippi (18). Other important differences include the source region and production stage of cattle sampled. A possible limitation of the present study is the evaluation of only one species of BRD pathogen cultured from NPS. Historically, *MH* has been the bacterial species most commonly isolated from the lungs of beef cattle that have died from respiratory disease and continues to be found frequently in cattle with respiratory disease (4, 57). However, ongoing studies of the upper respiratory tract microbiome suggest that other bacterial taxa are differentially abundant in cattle that go on to develop clinical BRD (26, 58). Care should be taken before concluding that AMR found in *MH* are predictive of AMR in other BRD associated microbes, or that these findings would be reflected in other beef cattle management situations. Antimicrobial susceptibility profiles have been shown to differ across geographic regions and may vary among bacterial species isolated from the same animal (11, 12, 22, 57). The selection of only one *MH* colony for some analyses may be considered a limitation of the present study. However, in 2022, Carter et al. showed that there was little phenotypic or genotypic variation in *MH* colonies isolated from NPS from an individual animal, and MICs differed by only one dilution factor when differences occurred (59); therefore, we considered the selection of only one colony for AST and WGS acceptable.

The administration of antimicrobials other than tulathromycin for BRD treatment was also associated with increased risk of isolation of MDR *MH* at 3 weeks after arrival. However, the magnitude of this effect differed depending on whether animals had fever at arrival; MDR *MH* was more likely to be isolated after BRD treatment from cattle that were febrile at arrival (Table 4). This suggests that timing of disease onset is an important factor in isolation of MDR *MH*, and these findings agree with a large study examining risk factors for isolation of *MH* and MDR *MH* from Canadian feedlot cattle (11). In this study, Noyes et al. found that, while any parenteral antimicrobial administration to an individual within 7 days of sampling was associated with decreased risk of isolation of *MH* from that animal, being in a pen with animals that had received parenteral antimicrobial at any time increased risk of isolation of *MH* and MDR *MH* (11). This suggests that, though antimicrobial treatment reduces isolation of *MH* for a short period of time, MDR *MH* may be spread to penmates of treated animals later. In the current study, until week 3 animals were separated into pastures based on metaphylaxis and antimicrobial treatment to prevent spread of *MH* that had been exposed to different antimicrobial drugs than other cattle in the group; therefore, we could not separate the effect of antimicrobial exposure in an individual from antimicrobial exposure of penmates. Spread of MDR *MH* from cattle that had been treated with AM to cattle that had not been treated could have influenced the WK10 results, as all animals were commingled after the WK3 sampling. However, the odds of isolation of MDR *MH* was still higher in animals in the META cattle even at WK10. One might expect no effect of TxGroup on odds of isolation of MDR *MH* if resistant bacteria were being spread among penmates. Though antimicrobial therapy for BRD had a lesser effect on the odds of recovery of MDR *MH* than metaphylaxis, this may be due to the difference in number of animals treated for BRD compared to those that received metaphylaxis.

### 4.2 Metaphylaxis improved weight gain of stocker calves

Interestingly, despite high prevalence of MDR *MH*, metaphylaxis remained effective at reducing overall and BRD morbidity (Tables 6, 8). Lack of blinding of pen riders to TxGroup is a significant consideration when interpreting BRD-related outcomes in the present study, and the results should be interpreted with this in mind. Diagnosis of BRD is currently based mainly on clinical signs, which is inherently subjective with limited sensitivity and specificity (5, 60). Despite lack of blinding, metaphylaxis reduced BRD-specific morbidity by approximately 50%, consistent with previous studies (8, 61). Weight gain is a more objective measure of animal wellbeing, and animals that received metaphylaxis gained more weight over a 3-week period than cattle that did not (Table 4). This effect was maintained over 10 weeks. Though there was an overall improvement in animal health in animals that received metaphylaxis, there was significant variability among Trials on the magnitude of that effect (Tables 5, 7). Specifically, Spring of 2021 had the highest crude and BRD morbidity and crude mortality of all Trials. Seasonality and weather have been described as contributors to BRD (62); however, it is not possible to parse any season effect on these outcomes in the present study, as this was the only spring Trial, which is another limitation of the present study. It is also of note that, in Spring 2021, an animal in the NO META TxGroup was BVD-PI positive, and this was the study in which results of BVD-PI testing were delayed, causing the PI animal to be left with the group for a few days. Bovine viral diarrhea virus is associated with immunosuppression and BRD, especially in conjunction with *MH* (3, 63–65). Therefore increased risk of BRD in the Spring 2021 Trial due to transient BVD exposure through the BVD-PI animal cannot be ruled out (66). Conversely, there was no effect of metaphylaxis on health in Fall 2019, which had the lowest morbidity. This highlights the variability among Trials. The variability in disease burden and the subjectivity of diagnosis is an inherent challenge in BRD research in stocker cattle.

Though we recognize this as a limitation of the study, the lack of blinding of pen riders was judged to be acceptable because the primary outcomes of interest, isolation of *MH* and MDR *MH* in cattle that did or did not receive metaphylaxis, were not influenced by decisions made by pen riders, and evaluating morbidity and mortality was a secondary objective of this study. Confirmation of *MH* culture, AST, and WGS was carried out by individuals who were blinded to TxGroup.

One possible reason for the continued efficacy of tulathromycin metaphylaxis for prevention of BRD in this trial, despite the high prevalence of tulathromycin resistance in isolated *MH*, could be a non-antimicrobial mechanism of action. Non-antimicrobial mechanisms of actions have been studied for multiple antimicrobial classes, including macrolides (67). Tulathromycin specifically has been shown to have inflammation modulation effects in cattle, by increasing pro-resolving mediators and lipoxins (68). This is especially interesting, because upregulation of gene expression related to lipoxin production has been shown to differentiate stocker calves that remain healthy compared to those that have severe BRD (69). As markers of inflammation or host gene expression data were not collected, no strong conclusions about potential non-antimicrobial effects of tulathromycin metaphylaxis can be made. It is also of note that *in vitro* susceptibility is not the same a clinical susceptibility *per se*. Timing of treatment, coinfection with multiple organisms, and host immunity are all important factors in determining treatment success (3, 60, 70). In order to determine the effect outside of *MH*, future work evaluating the effect of metaphylaxis on the nasopharyngeal microbiome and resistome, similar to what has been done in feces (21), is warranted.

### 4.3 Antimicrobial resistance is associated with mobile genetic elements

Antimicrobial resistance in the present study appeared to be largely mediated by ICE (Figures 2, 3, 5, Table 4, and Table S9). All resistance genes identified here are have been identified in previously described ICE (14, 16, 71). Since the discovery of ICE*Pmu1*, there has been increasing interest in the role of ICE in AMR and MDR in BRD pathogens. Described ICE contain a core backbone maintained through replication that harbors genes necessary for conjugation, transfer between bacteria, and integration into the bacterial host genome. More variation is seen in ICE when observing the ARG regions, with some ICE containing no ARG (16). The findings presented here are consistent with that variation in ICE-associated ARGs. There were 15 different ARG profiles identified in 108 MH isolates with ICE-associated genes. It is possible that ARGs identified in the present study are not contained in ICE, though this seems less likely due to the strong relationship between ICE presence and MDR in these isolates, and ARG patterns associated with previously described ICE (4, 14, 16, 71). The presence of multiple ARGs on one mobile genetic element has important implications for treatment options, as there are limited antimicrobial classes labeled for BRD treatment. ARGs to all of these classes have been previously associated with ICE found in *Pasteurellaceae*. Previous work with *MH* and *P. multocida* transconjugants containing an ICE with multiple ARGs showed that there were no synergistic combinations of antimicrobials approved for BRD treatment on these isolates *in vitro*, and that ICE were maintained in these transconjugants after long-term passage, despite a fitness cost (71). These findings highlight the difficulty AMR could cause for veterinarians and producers, and the importance of investigating antimicrobial alternatives for treating BRD. It is also important to note that ICE-associated genes were found in isolates across the range of genetic diversity described herein (Figure 6), although the diversity was limited, with the majority of MDR isolates occurring within one genetic cluster. Previous research has produced differing results when describing the genetic variability of MDR *MH* (15, 18). Woolums et al. (18) described high genetic variability among isolates characterized by pulse field gel electrophoresis in MDR *MH* isolated 3 weeks after arrival. In contrast, the current results agree more with previous work by Snyder et al. (15) which evaluated WGS of MDR *MH* isolated from stocker calves 2 weeks after arrival, where all 2-week *MH* isolates were in one clade. While not all *MH* isolates collected at a time after arrival were in one cluster, genetic variability was decreased (Figure 6). This clustering was similar between META and NO META TxGroups indicating this decrease in genetic diversity may be inherent to the process of backgrounding stocker calves, as has been previously suggested (15).

## 5 Conclusion

Tulathromycin metaphylaxis was associated with increased isolation of MDR *MH* while improving health in stocker calves. Post-arrival antimicrobial treatment for clinical BRD was also associated with increased isolation of MDR *MH* in cattle that were febrile at arrival. The resistance identified in the present study seemed to be largely driven by antimicrobial resistance genes contained in ICE. Further research is needed to address the effect of metaphylaxis on the nasopharyngeal microbial communities and ARGs found in other, uncultured bacterial species, and to investigate antimicrobial alternatives to help control BRD, as removing metaphylaxis could have serious animal welfare impacts.

## Data availability statement

The datasets presented in this study can be found as supplementary material or in online repositories. The names of the repository/repositories and accession number(s) can be found at: https://www.ncbi.nlm.nih.gov/genbank/, SAMN33700459- SAMN33700763; https://www.ncbi.nlm.nih.gov/sra, SAMN33700459- SAMN33700763.

## Ethics statement

The animal study was approved by Mississippi State University Institutional Animal Care and Use Committee. The study was conducted in accordance with the local legislation and institutional requirements.

## Author contributions

WC: Data curation, Formal analysis, Investigation, Methodology, Writing – original draft, Writing – review & editing. BK: Conceptualization, Data curation, Formal analysis, Funding acquisition, Investigation, Methodology, Resources, Supervision, Writing – review & editing. LH: Data curation, Formal analysis, Investigation, Methodology, Resources, Software, Writing – review & editing. LP: Formal analysis, Investigation, Methodology, Resources, Software, Writing – review & editing. AP: Data curation, Investigation, Methodology, Resources, Writing – review & editing. JF: Conceptualization, Data curation, Formal analysis, Funding acquisition, Investigation, Methodology, Resources, Software, Writing – review & editing. CJ: Conceptualization, Formal analysis, Funding acquisition, Investigation, Methodology, Resources, Supervision, Writing – review & editing. JL: Formal analysis, Investigation, Methodology, Resources, Writing – review & editing. WE: Conceptualization, Formal analysis, Funding acquisition, Investigation, Resources, Supervision, Writing – review & editing. JB: Investigation, Methodology, Project administration, Resources, Supervision, Writing – review & editing, Conceptualization. SC: Conceptualization, Data curation, Formal analysis, Investigation, Methodology, Resources, Writing – review & editing. PM: Conceptualization, Data curation, Formal analysis, Funding acquisition, Investigation, Methodology, Resources, Software, Supervision, Writing – review & editing. AW: Conceptualization, Data curation, Formal analysis, Funding acquisition, Investigation, Methodology, Project administration, Resources, Supervision, Writing – review & editing.
