# Supplementary material for: Tulathromycin metaphylaxis increases nasopharyngeal isolation of multidrug resistant Mannheimia haemolytica in stocker heifers
Source: Front Vet Sci. 2023 Nov 20;10:1256997. doi: 10.3389/fvets.2023.1256997 (PMC10694364; doi:10.3389/fvets.2023.1256997)
Supplement: Supplementary file 1 [file Data_Sheet_1.zip › Datasheet S1.pdf]

Processing Protocol:

Day 0: weigh, ear tag for ID, rectal temperature, vaccinate with Pyramid 5 and Vision 7 with SPUR, deworm with fenbendazole orally (Safeguard) and doramectin by injection (Dectomax). Nasopharyngeal swab (NPS) x 4--two from each nostril, ear notch, draw blood (1 red top for serum). Metaphylaxis per treatment group, move to pastures.

Ear notches to be sent to Pearl Diagnostic lab for testing the next day; any cattle found positive to be removed from study.

Day 6: weigh

Day 13: weigh

Day 20: weigh, draw blood (1 red top), NPS x 4, booster vaccines

After Day 20, cattle to be comingled on ryegrass.

Day 69: weigh, draw blood (1 red top back in 2 hours), NPS x 4

Deworming Protocol-

Safeguard (fenbendazole, Merck Animal Health) given orally to all animals at approximately 550 lb dose (13 mL).
Withdrawal: 8 days from the last day of administration

Dectomax (Doromectin, Zoetis Inc.)
Dosage: *Administer as a single subcutaneous injection in the neck at dosage of 10 mg/kg.* ***(1 mL per 110 lb BW)***

Dosage by bodyweight for Dectomax (SC in neck)

330-439 lbs	440-549 lbs	550 -659 lbs	660-769 lbs
4 mL	5 mL	6 mL	7 mL

Withdrawal time: 35 days from the last day of administration
