# Supplementary material for: Tulathromycin metaphylaxis increases nasopharyngeal isolation of multidrug resistant Mannheimia haemolytica in stocker heifers
Source: Front Vet Sci. 2023 Nov 20;10:1256997. doi: 10.3389/fvets.2023.1256997 (PMC10694364; doi:10.3389/fvets.2023.1256997)
Supplement: Supplementary file 1 [file Data_Sheet_1.zip › Datasheet S2.pdf]

<u>BRD Scoring system (Adapted From Step et al, 2008)</u>

0 = Normal

1 = Mild BRD including one or more of the following signs:

- elevated respiratory rate for the environmental conditions
- mild to moderate gauntness
- mild depressed attitude: not as alert as expected when viewed from a distance
  becomes alert when animal sees human observer
- shallow or dry cough

Cattle with score of 1 may also have cloudy, white, or yellow nasal discharge.
Nasal discharge in the absence of any other abnormalities is not enough for a score of 1.

2 = Moderate BRD including one or more of the following signs:

- mild or moderate depression
  - lethargic, but may look alert when approached
  - head carriage lower than normal, but returns to normal when approached
  - hiding behavior: tends to stay behind other cattle, relative to the observer
- mild to moderate muscle weakness
  - stepping slowly when walking, or mild incoordination
  - droopy ears
- repeated coughing
- moderate gauntness
- breathing with mild to moderately increased abdominal effort

Cattle with a score of 2 may also have:
elevated respiratory rate for environmental conditions
clear, cloudy, white, or yellow nasal discharge.

3 = Severe BRD including one or more of the following signs:

- severe depression or weakness
  - lethargic and does not look more alert when approached
  - low head carriage, does not return to normal when approached
  - does not move away from examiner as expected when approached
  - cross stepping
- Repeated deep cough
- Severe breathing effort
  - open mouth breathing or panting
  - moderately to markedly increased abdominal effort

Cattle with a score of 3 may also have:

elevated respiratory rate for the environmental conditions
clear, cloudy, white, or yellow nasal discharge
and/or moderate to extreme gauntness.

4 = Moribund (near death)

- recumbent and does not rise when approached or directly stimulated

  OR

- standing but does not move unless directly stimulated
  - if the animal moves, it is very weak: drags feet, sways, stumbles, falls down
- eyes may be very sunken, abdomen may be very gaunt

Moribund animals may also have signs described for score of 1, 2, or 3.

Coughing may be heard from animals with any score.

NOTE: sometimes animals near death may act aggressively, trying to charge an observer

<u>BRD Case Definition</u>

BRD score = 1 or 2 AND have a rectal temperature ≥104 ºF

OR

a BRD score ≥ 3 regardless of rectal temperature.

WITH

no other obvious signs of disease (lameness, diarrhea, swollen legs, strange behavior, etc.)
