# Supplementary material for: Tulathromycin metaphylaxis increases nasopharyngeal isolation of multidrug resistant Mannheimia haemolytica in stocker heifers
Source: Front Vet Sci. 2023 Nov 20;10:1256997. doi: 10.3389/fvets.2023.1256997 (PMC10694364; doi:10.3389/fvets.2023.1256997)
Supplement: Supplementary file 1 [file Data_Sheet_1.zip › Datasheet S3.pdf]

BRD Treatment

First treatment

Excede (Ceftiofur Crystalline Free Acid, Zoetis Inc.)

Dosage: *Administer as a single subcutaneous injection in the posterior aspect of the ear where it attaches to the head (base of the ear) to cattle at a dosage of 3.0 mg ceftiofur equivalents (CE)/lb (6.6 mg CE/kg) body weight (BW)* ***(1.5 mL sterile suspension per 100 lb BW)***

Dosage by bodyweight for Excede (base of ear)

351-400 lbs	401-450 lbs	451 -500 lbs	501-550 lbs	551 – 600 lbs	601 – 650 lbs	651 – 700 lbs
6 ml	6.8 ml	7.5 ml	8.3 ml	9 ml	9.8 ml	10.5 ml

Post-treatment interval: 7 days (no additional treatments for BRD for 7 days after administering Excede)

Withdrawal time: 13 days from the last day of administration

Second treatment (retreatment if necessary 7 days after treatment with Excede)

Nuflor (Florfenicol, Merck Animal Health)

Dosage: *NUFLOR Injectable Solution can be administered by a single subcutaneous (SC) injection to cattle at a dose rate of 40 mg/kg body weight* ***(6 mL/100 lbs). Do not administer more than 10 mL at each site. The injection should be given only in the neck.***

Dosage by bodyweight for Nuflor (SC injection neck)

351-400 lbs	401-450 lbs	451 -500 lbs	501-550 lbs	551 – 600 lbs	601 – 650 lbs	651 – 700 lbs
24 ml	27 ml	30 ml	33 ml	36 ml	39 ml	42 ml

Post-treatment interval 4 days

Withdrawal time: 38 days from the last day of administration.

Third treatment (retreatment 4 days after treatment with Nuflor)

Noromycin 300 LA (oxytetracycline 300mg/ml, Norbrook Laboratories)

Dosage: *A single dosage of 20 mg/kg* ***(3 ml/100 lbs) of bodyweight administered under the skin of the neck . No more than 10 ml per injection site.***

Dosage by bodyweight for Noromycin 300 (SC neck)

351-400 lbs	401-450 lbs	451 -500 lbs	501-550 lbs	551 – 600 lbs	601 – 650 lbs	651 – 700 lbs
12 ml	13.5 ml	15 ml	16.5 ml	18 ml	19.5 ml	21 ml

Withdrawal time: 28 days from the last day of administration
