# Supplementary material for: Tulathromycin metaphylaxis increases nasopharyngeal isolation of multidrug resistant Mannheimia haemolytica in stocker heifers
Source: Front Vet Sci. 2023 Nov 20;10:1256997. doi: 10.3389/fvets.2023.1256997 (PMC10694364; doi:10.3389/fvets.2023.1256997)
Supplement: Supplementary file 1 [file Data_Sheet_1.zip › Table S1.docx]

**Table S1.** Genes used for determination of ICE presence

| Gene | Type | Locus Tag | Reference |
| --- | --- | --- | --- |
| *int1* | Integrase | Pmu_02700 | (45) |
| *int2* |  | Pmu_02880 | (45) |
| *rel1* | Relaxase | *Mh*H_c22640 | (14) |
| *rel2* |  | Pmu_02890 | (45) |
| *traC* | Transferase | Pmu_03070 | (45) |
| *traD* |  | Pmu_03190 | (45) |
| *traG* |  | Pmu_03040 | (45) |

**Legend:** Key: ICE, integrative-conjugative element; *int1,* tyrosine recombinase-1 family protein; *int2,* tyrosine recombinase-2 family protein; *rel1*, DNA-binding domain-containing protein; *rel2*, integrating conjugative element relaxase, PFGI-1 class; *traC*, conjugative transfer ATPase TraC-like, PFL family; *traD*, conjugal transfer protein TraD; *traG*, TraG-like domain-containing protein.
