# Supplementary material for: Tulathromycin metaphylaxis increases nasopharyngeal isolation of multidrug resistant Mannheimia haemolytica in stocker heifers
Source: Front Vet Sci. 2023 Nov 20;10:1256997. doi: 10.3389/fvets.2023.1256997 (PMC10694364; doi:10.3389/fvets.2023.1256997)
Supplement: Supplementary file 1 [file Data_Sheet_1.zip › Table S10.docx]

**Table S10.** Univariable models for *Mannheimia haemolytica* isolation outcomes at 3 weeks

| Input | **Outcome** | | | | | |
| --- | --- | --- | --- | --- | --- | --- |
|  | *MH* Isolation | P-value | MDR *MH* Isolation | P-value | ICE Presence | P-value |
| Group^#^ | | | | | | |
| META | Ref | Ref | Ref | Ref | Ref | Ref |
| NO META | 0.71 (0.45-1.11) | 0.13* | 0.14 (0.07-0.28) | <0.0001* | 0.29 (0.18-0.48) | <0.0001* |
| Fever at Arrival | | | | | | |
| Yes | Ref | Ref | Ref | Ref | Ref | Ref |
| No | 1.97 (0.96-4.02) | 0.06* | 2.28  (0.95-5.43) | 0.06* | 2.02 (0.93-4.39) | 0.07* |
| Weight at arrival | | | | | | |
| 232 kg | Ref | Ref | Ref | Ref | Ref | Ref |
| Difference (kg) | 1.001 (1.00-1.02) | 0.015* | 0.99  (0.98-1.02) | 0.94 | 1.00 (0.99-1.02) | 0.54 |
| *MH* Isolation at arrival | | | | | | |
| Yes | Ref | Ref | Ref | Ref | Ref | Ref |
| No | 1.13 (0.64-2.01) | 0.67 | 1.36 (0.67-2.77) | 0.39 | 1.39 (0.76-2.52) | 0.28 |
| MDR *MH* isolation at arrival | | | | | | |
| Yes | Ref | Ref | Ref | Ref | Ref | Ref |
| No | 0.67 (0.23-1.94) | 0.47 | 0.71 (0.23-2.12) | 0.54 | 0.62 (0.22-1.71) | 0.35 |
| ICE presence in *MH* at arrival | | | | | | |
| Yes | Ref | Ref | Ref | Ref | Ref | Ref |
| No | 0.62 (0.23-1.66) | 0.34 | 0.56  (0.21-1.52) | 0.34 | 0.66 (0.26-1.69) | 0.39 |
| Isolation of genotype 2 *MH* at arrival | | | | | | |
| Yes | Ref | Ref | Ref | Ref | Ref | Ref |
| No | 1.10 (0.54-2.25) | 0.79 | 1.32  (0.58-3.02) | 0.50 | 1.56 (0.73-3.32) | 0.25 |
| BRD treatment at 3 weeks | | | | | | |
| Yes | Ref | Ref | Ref | Ref | Ref | Ref |
| No | 1.05 (0.58-1.88) | 0.87 | 0.57  (0.28-1.16) | <0.0001* | 1.01 (0.55-1.86) | 0.96 |

**Legend:**BRD treatment (Yes or No) indicates whether an animal received antimicrobials for BRD treatment before 3 week sampling. ^#^Group (META or NO META) was included in all multivariable models, regardless of *P*-value. Weight at arrival input is difference from median weight (232 kg). Abreviations: BRD, bovine respiratory disease;; MDR, multidrug resistant; Ref, reference; OR, Odds Ratio; CI, confidence interval. *Variable was eligible for inclusion in final multivariable model (*P<*0.2).
