# Supplementary material for: Tulathromycin metaphylaxis increases nasopharyngeal isolation of multidrug resistant Mannheimia haemolytica in stocker heifers
Source: Front Vet Sci. 2023 Nov 20;10:1256997. doi: 10.3389/fvets.2023.1256997 (PMC10694364; doi:10.3389/fvets.2023.1256997)
Supplement: Supplementary file 1 [file Data_Sheet_1.zip › Table S11.docx]

**Table S11.** Univariable models for *Mannheimia haemolytica* isolation outcomes at 10 weeks

| Input | | **Outcome** | | | | | | | | | |
| --- | --- | --- | --- | --- | --- | --- | --- | --- | --- | --- | --- |
|  |  | *MH* Isolation | | P-value | MDR *MH* Isolation | | P-value | | | ICE Presence | P-value |
| Group^#^ | | | | | | | | | | | |
| META | | Ref | | Ref | Ref | | Ref | | | Ref | Ref |
| NO META | | 0.57 (0.28-1.19) | | 0.14* | 0.17 (0.04-0.75) | | 0.019* | | | 0.53 (0.20-1.44) | 0.21* |
| Fever at Arrival | | | | | | | | | | | |
| Yes | | Ref | | Ref | Ref | | Ref | | | Ref | Ref |
| No | | 0.50 (0.20-1.25) | | 0.14* | 0.69 (0.18-2.62) | | 0.58 | | | 0.90 (0.24-3.32) | 0.87 |
| Weight at arrival | | | | | | | | | | | |
| 232 kg | | Ref | | Ref | Ref | | Ref | | | Ref | Ref |
| Difference (kg) | | 0.99 (0.98-1.02) | | 0.64 | 1.00 (0.98-1.03) | | 0.76 | | | 0.99 (0.96-1.02) | 0.54 |
| *MH* Isolation at Arrival | | | | | | | | | | | |
| Yes | Ref | | Ref | | | Ref | | Ref | Ref | | Ref |
| No | **2.30 (0.28-1.19)** | | **0.14*** | | | **3.06 (0.39-24.00)** | | **0.29** | **4.70 (0.61-36.25)** | | 0.14* |
| MDR MH isolation at Arrival | | | | | | | | | | | |
| Yes | **Models Failed to Converge** | | | | | | | | | | |
| No |  |  |  |  |  |  |  |  |  |  |  |
| ICE Presence in MH at Arrival | | | | | | | | | | | |
| Yes | **Models Failed to Converge** | | | | | | | | | | |
| No |  |  |  |  |  |  |  |  |  |  |  |
| Isolation of genotype 2 *MH* at Arrival | | | | | | | | | | | |
| Yes | **Ref** | | ***Ref*** | | | ***Models Failed to Converge*** | | | | | |
| No | **5.97 (0.78-45.56)** | | **0.08*** | | |  |  |  |  |  |  |
| *MH* Isolation at Week 3 | | | | | | | | | | | |
| Yes | | Ref | | Ref | Ref | | Ref | | | Ref | Ref |
| No | | 1.13 (0.55-2.33) | | 0.74 | 0.84 (0.28-2.50) | | 0.75 | | | 1.05 (0.39-2.82) | 0.92 |
| MDR *MH* isolation at Week 3 | | | | | | | | | | | |
| Yes | | Ref | | Ref | Ref | | Ref | | | Ref | Ref |
| No | | 0.59 (0.26-1.30) | | 0.19* | 0.36 (0.12-1.12) | | 0.08* | | | 0.54 (0.19-1.55) | 0.25 |
| ICE presence in *MH* at Week 3 | | | | | | | | | | | |
| Yes | | Ref | | Ref | Ref | | Ref | | | Ref | Ref |
| No | | 0.88 (0.42-1.88) | | 0.76 | 0.52 (0.17-1.54) | | 0.24 | | | 0.65 (0.24-1.74) | 0.39 |
| Isolation of genotype 2 *MH* at Week 3 | | | | | | | | | | | |
| Yes | | Ref | | Ref | Ref | | Ref | | | Ref | Ref |
| No | | 1.11 (0.54-2.30) | | 0.77 | 0.83 (0.28-2.47) | | 0.73 | | | 1.04 (0.39-2.78) | 0.94 |
| BRD treatment at 10 weeks | | | | | | | | | | | |
| Yes | | Ref | | Ref | Ref | | Ref | | | Ref | Ref |
| No | | 1.78 (0.60-5.28) | | 0.30 | 1.22 (0.27-5.50) | | 0.80 | | | 0.69 (0.22-2.19) | 0.53 |

**Legend:**BRD treatment (Yes or No) indicates whether an animal received antimicrobials for BRD treatment before 10 week sampling. ^#^TxGroup (META or NO META) was included in all multivariable models, regardless of *P*-value. Weight at arrival input is difference from median weight (232 kg). Abreviations: BRD, bovine respiratory disease;; MDR, multidrug resistant; Ref, reference; OR, Odds Ratio; CI, confidence interval. *Variable was eligible for inclusion in final multivariable model (*P<*0.2).
