# Supplementary material for: Tulathromycin metaphylaxis increases nasopharyngeal isolation of multidrug resistant Mannheimia haemolytica in stocker heifers
Source: Front Vet Sci. 2023 Nov 20;10:1256997. doi: 10.3389/fvets.2023.1256997 (PMC10694364; doi:10.3389/fvets.2023.1256997)
Supplement: Supplementary file 1 [file Data_Sheet_1.zip › Table S12.docx]

**Table S12.** Univariable models for animal health outcomes at 3 weeks

| Input | **Outcome** | | | | | |
| --- | --- | --- | --- | --- | --- | --- |
|  | OR BRD Morbidity (95% CI) | P-value | OR Mortality  (95% CI) | P-value | Est. ADG  (SE) | P-value |
| Group^#^ | | | | | | |
| META | Ref | Ref | Ref | Ref | Ref | Ref |
| NO META | 2.86  (1.61-5.12) | 0.004* | 2.50  (0.63-9.96) | 0.19* | -0.36 (0.08) | <0.0001* |
| Fever at Arrival | | | | | | |
| Yes | Ref | Ref | Ref | Ref | Ref | Ref |
| No | 0.96  (0.44-2.12) | 0.927 | 1.83  (0.22-15.35) | 0.58 | 0.35(0.13) | 0.007* |
| Weight at arrival | | | | | | |
| 232 kg | Ref | Ref | Ref | Ref | Ref | Ref |
| Difference (kg) | 0.97  (0.96-0.99) | 0.011* | 0.98  (0.94-1.02) | 0.33 | 0.003  (0.002) | 0.16* |
| *MH* Isolation at arrival | | | | | | |
| Yes | Ref | Ref | Ref | Ref | Ref | Ref |
| No | 0.75  (0.40-1.41) | 0.38 | 0.44  (0.10-2.00) | 0.44 | -0.06  (0.11) | 0.59 |
| MDR *MH* isolation at arrival | | | | | | |
| Yes | Ref | Ref | Ref | Ref | Ref | Ref |
| No | 0.58  (0.20-1.70) | 0.32 | 0.28  (0.03-3.07) | 0.30 | 0.01  (0.20) | 0.98 |
| ICE presence in *MH* at arrival | | | | | | |
| Yes | Ref | Ref | Ref | Ref | Ref | Ref |
| No | 0.60  (0.22-1.61) | 0.31 | 0.14  (0.021-0.96) | 0.046* | 0.04  (0.19) | 0.84 |
| Isolation of genotype 2 *MH* at arrival | | | | | | |
| Yes | Ref | Ref | Ref | Ref | Ref | Ref |
| No | 0.90  (0.41-1.99) | 0.80 | 0.40  (0.07-2.28) | 0.30 | -0.04  (0.14) | 0.80 |
| BRD treatment at 3 weeks | | | | | | |
| Yes | Ref | Ref | Ref | Ref | Ref | Ref |
| No | N/A | N/A | 17.54  (17.4-17.7) | <0.0001* | 0.98  (0.10) | <0.0001* |

**Legend:** BRD Morbidity is the number of animals treated at least once for BRD over first 3 weeks of study period. Mortality includes all animals who died over the 3 week study period. ^#^Group (META or NO META) was included in all multivariable models, regardless of *P*-value. Weight at arrival input is difference from median weight (232 kg). Abbreviations: BRD, bovine respiratory disease; ADG, average daily gain (kg/day); *MH, Mannheimia haemolytica*; MDR, multidrug resistant; ICE, integrative conjugative element; Ref, reference; OR, Odds Ratio; CI, confidence interval; SE, standard error; Est, Restricted Maximum Likelihood estimate. *Variable was eligible for inclusion in final multivariable model (*P<*0.2).
