# Supplementary material for: Tulathromycin metaphylaxis increases nasopharyngeal isolation of multidrug resistant Mannheimia haemolytica in stocker heifers
Source: Front Vet Sci. 2023 Nov 20;10:1256997. doi: 10.3389/fvets.2023.1256997 (PMC10694364; doi:10.3389/fvets.2023.1256997)
Supplement: Supplementary file 1 [file Data_Sheet_1.zip › Table S13.docx]

**Table S13.** Univariable models for animal health outcomes at 10 weeks

| Input | **Outcome** | | | | | |
| --- | --- | --- | --- | --- | --- | --- |
|  | BRD Morbidity | P-value | Mortality | P-value | ADG | P-value |
| Group | | | | | | |
| META | Ref | Ref | Ref | Ref | Ref | Ref |
| NO META | 2.61 (1.51-4.51) | 0.0006* | 2.02 (0.73-5.57) | 0.17* | -0.21 (0.08) | 0.01* |
| Fever at Arrival | | | | | | |
| Yes | Ref | Ref | Ref | Ref | Ref | Ref |
| No | 1.14 (0.52-2.49) | 0.75 | 0.66 (0.20-2.16) | 0.49 | 0.19 (0.12) | 0.11* |
| Weight at arrival | | | | | | |
| 232 kg | Ref | Ref | Ref | Ref | Ref | Ref |
| Difference (kg) | 0.98 (0.97-0.99) | 0.02* | 0.99 (0.96-1.02) | 0.74 | 0.003 (0.002) | 0.14* |
| *MH* Isolation at arrival | | | | | | |
| Yes | Ref | Ref | Ref | Ref | Ref | Ref |
| No | 0.68 (0.37-1.24) | 0.21 | 0.18 (0.17-0.18) | <0.0001* | -0.04 (0.10) | 0.72 |
| MDR *MH* isolation at arrival | | | | | | |
| Yes | Ref | Ref | Ref | Ref | Ref | Ref |
| No | 0.68 (0.23-1.98) | 0.48 | 0.16 (0.03-0.97) | 0.046* | 0.04 (0.18) | 0.82 |
| ICE presence in *MH* at arrival | | | | | | |
| Yes | Ref | Ref | Ref | Ref | Ref | Ref |
| No | 0.70 (0.26-1.88) | 0.48 | 0.13 (0.03-0.64) | 0.011* | 0.03 (0.17) | 0.87 |
| Isolation of genotype 2 *MH* at arrival | | | | | | |
| Yes | Ref | Ref | Ref | Ref | Ref | Ref |
| No | 0.91 (0.42-1.95) | 0.81 | 0.24 (0.06-0.92) | 0.037* | -0.11 (0.12) | 0.36 |
| MH Isolation at week 3 | | | | | | |
| Yes | Ref | Ref | Ref | Ref | Ref | Ref |
| No | 1.11 (0.64-1.93) | 0.70 | 0.43 (0.09-2.08) | 0.29 | 0.005 (0.08) | 0.95 |
| MDR MH isolation at week 3 | | | | | | |
| Yes | Ref | Ref | Ref | Ref | Ref | Ref |
| No | 0.65 (0.35-1.21) | 0.18* | 0.35 (0.07-1.71) | 0.20* | 0.08 (0.1) | 0.41 |
| ICE presence in MH at week 3 | | | | | | |
| Yes | Ref | Ref | Ref | Ref | Ref | Ref |
| No | 1.07 (0.60-1.91) | 0.82 | 0.29 (0.06-1.39) | 0.12* | -0.02 (0.08) | 0.8 |
| Isolation of genotype 2 MH at week 3 | | | | | | |
| Yes | Ref | Ref | Ref | Ref | Ref | Ref |
| No | 1.10 (0.63-1.90) | 0.74 | 0.42 (0.09-2.06) | 0.29 | 0.01 (0.08) | 0.92 |
| BRD treatment at 10 weeks | | | | | | |
| Yes | Ref | Ref | Ref | Ref | Ref | Ref |
| No | N/A | N/A | 0.05 (0.01-0.17) | <0.0001 | 0.52 (0.09) | <0.0001 |

**Legend:** BRD Morbidity is the number of animals treated at least once for BRD over first 3 weeks of study period. Mortality includes all animals who died within 3 weeks of arrival. ^#^Group (META or NO META) was included in all multivariable models, regardless of *P*-value. Weight at arrival input is difference from median weight (232 kg). Abbreviations: BRD, bovine respiratory disease; ADG, average daily gain (kg/day); *MH, Mannheimia haemolytica*; MDR, multidrug resistant; ICE, integrative conjugative element; Ref, reference; OR, Odds Ratio; CI, confidence interval; SE, standard error; Est, Restricted Maximum Likelihood estimate. *Variable was eligible for inclusion in final multivariable model (*P<*0.2).
