# Supplementary material for: Tulathromycin metaphylaxis increases nasopharyngeal isolation of multidrug resistant Mannheimia haemolytica in stocker heifers
Source: Front Vet Sci. 2023 Nov 20;10:1256997. doi: 10.3389/fvets.2023.1256997 (PMC10694364; doi:10.3389/fvets.2023.1256997)
Supplement: Supplementary file 1 [file Data_Sheet_1.zip › Table S2.docx]

**Table S2. Factors tested in regression models**

| Outcome Variable (Time) | Modeling Type | Input Variables | Input Variable Type (Outcome/Unit) |
| --- | --- | --- | --- |
| Isolation of *Mh* (3WK) | Logistic | Arrival *Mh*  Arrival MDR *Mh*  Arrival Genotype 2 *Mh*  Prior BRD treatment  Arrival weight  Fever at Arrival | Categorical (Yes, No)  Categorical (Yes, No)  Categorical (Yes, No)  Categorical (Yes, No)  Continuous (kg)  Categorical (Yes, No) |
| Isolation of MDR *Mh* (3WK) | Logistic | Arrival *Mh*  Arrival MDR *Mh*  Arrival Genotype 2 *Mh*  Prior BRD treatment  Arrival weight  Fever at Arrival | Categorical (Yes, No)  Categorical (Yes, No)  Categorical (Yes, No)  Categorical (Yes, No)  Continuous (kg)  Categorical (Yes, No) |
| Isolation of *Mh* with ICE (3WK) | Logistic | Arrival *Mh*  Arrival MDR *Mh*  Arrival Genotype 2 *Mh*  Prior BRD treatment  Arrival weight  Fever at Arrival | Categorical (Yes, No)  Categorical (Yes, No)  Categorical (Yes, No)  Categorical (Yes, No)  Continuous (kg)  Categorical (Yes, No) |
| Animals Treated for BRD (3WK) | Logistic | Arrival *Mh*  Arrival MDR *Mh*  Arrival Genotype 2 *Mh*  Arrival weight  Fever at Arrival | Categorical (Yes, No)  Categorical (Yes, No)  Categorical (Yes, No)  Continuous (kg)  Categorical (Yes, No) |
| Animals Died (3WK) | Logistic | Arrival *Mh*  Arrival MDR *Mh*  Arrival Genotype 2 *Mh*  Prior BRD treatment  Arrival weight  Fever at Arrival | Categorical (Yes, No)  Categorical (Yes, No)  Categorical (Yes, No)  Categorical (Yes, No)  Continuous (kg)  Categorical (Yes, No) |
| ADG (3WK) | Linear | Arrival *Mh*  Arrival MDR *Mh*  Arrival Genotype 2 *Mh*  Prior BRD treatment  Arrival weight  Fever at Arrival | Categorical (Yes, No)  Categorical (Yes, No)  Categorical (Yes, No)  Categorical (Yes, No)  Continuous (kg)  Categorical (Yes, No) |
| Isolation of *Mh* (10WK) | Logistic | Arrival or WK3 *Mh*  Arrival and WK3 MDR *Mh*  Arrival and WK3 Genotype 2 *Mh*  Prior BRD treatment  Arrival weight  Fever at Arrival | Categorical (Yes, No)  Categorical (Yes, No)  Categorical (Yes, No)  Categorical (Yes, No)  Continuous (kg)  Categorical (Yes, No) |
| Isolation of MDR *Mh* (10WK) | Logistic | Arrival or WK3 *Mh*  Arrival or WK3 MDR *Mh*  Arrival or WK3 Genotype 2 *Mh*  Prior BRD treatment  Arrival weight  Fever at Arrival | Categorical (Yes, No)  Categorical (Yes, No)  Categorical (Yes, No)  Categorical (Yes, No)  Continuous (kg)  Categorical (Yes, No) |
| Isolation of *Mh* with ICE (10WK) | Logistic | Arrival or WK3 *Mh*  Arrival or WK3 MDR *Mh*  Arrival or WK3 Genotype 2 *Mh*  Prior BRD treatment  Arrival weight  Fever at Arrival | Categorical (Yes, No)  Categorical (Yes, No)  Categorical (Yes, No)  Categorical (Yes, No)  Continuous (kg)  Categorical (Yes, No) |
| Animals Treated for BRD (10WK) | Logistic | Arrival or 3WK *Mh*  Arrival or 3WK MDR *Mh*  Arrival or 3WK Genotype 2 *Mh*  Prior BRD treatment  Arrival weight  Fever at Arrival | Categorical (Yes, No)  Categorical (Yes, No)  Categorical (Yes, No)  Categorical (Yes, No)  Continuous (kg)  Categorical (Yes, No) |
| Animals Died (10WK) | Logistic | Arrival or 3WK *Mh*  Arrival or 3WK MDR *Mh*  Arrival or WK3 ICE *Mh*  Arrival or 3WK Genotype 2 *Mh*  Prior BRD treatment  Arrival weight | Categorical (Yes, No)  Categorical (Yes, No)  Categorical (Yes, No)  Categorical (Yes, No)  Categorical (Yes, No)  Continuous (kg) |
| ADG (10WK) | Linear | Arrival or 3WK *Mh*  Arrival or 3WK MDR *Mh*  Arrival or 3WK ICE *Mh*  Arrival or 3WK Genotype 2 *Mh*  Prior BRD treatment  Arrival weight | Categorical (Yes, No)  Categorical (Yes, No)  Categorical (Yes, No)  Categorical (Yes, No)  Categorical (Yes, No)  Continuous (kg) |
