# Supplementary material for: Tulathromycin metaphylaxis increases nasopharyngeal isolation of multidrug resistant Mannheimia haemolytica in stocker heifers
Source: Front Vet Sci. 2023 Nov 20;10:1256997. doi: 10.3389/fvets.2023.1256997 (PMC10694364; doi:10.3389/fvets.2023.1256997)
Supplement: Supplementary file 1 [file Data_Sheet_1.zip › Table S7.docx]

**Table S7. Agreement between phenotypic and genotypic identification of antimicrobial resisistance**

| Conc | Pattern  (AST,ARG) | Amin* | % | B-lac | % | MLS | % | Phen* | % | Tet | % |
| --- | --- | --- | --- | --- | --- | --- | --- | --- | --- | --- | --- |
| Agree | YY | 71 | 82 | 115 | 89 | 115 | 93 | 69 | 97 | 175 | 95 |
|  | NN | 178 |  | 156 |  | 170 |  | 226 |  | 116 |  |
| Disagree | YN | 52 | 18 | 20 | 11 | 12 | 6.6 | 6 | 3.3 | 7 | 4.6 |
|  | NY | 4 |  | 14 |  | 8 |  | 4 |  | 7 |  |

**Legend:** Concordance of antimicrobial resistance measurements. *There was a statisitically significant difference between ARG and AST in classifying an isolate as resitant to antimicrobial class (McNemar’s χ^2^ test, *P*<0.05). Key: AST, antimicrobial susceptibility testing; Gen, antimicrobial resistance genes; Amin, aminoglycosides; B-lac, beta-lactams; MLS, macrolides-lincosamides-streptogramins; Phen, phenicols; Tet, tetracyclines; Y, not susceptible or antimicrobial resistance gene found; N, susceptible or antimicrobial resistance genes not found.
