# Supplementary material for: Tulathromycin metaphylaxis increases nasopharyngeal isolation of multidrug resistant Mannheimia haemolytica in stocker heifers
Source: Front Vet Sci. 2023 Nov 20;10:1256997. doi: 10.3389/fvets.2023.1256997 (PMC10694364; doi:10.3389/fvets.2023.1256997)
Supplement: Supplementary file 1 [file Data_Sheet_1.zip › Table S8.docx]

**Table S8:** *Mannheimia haemolytica* isolation at arrival, week 3, and week 10 across 4 separate trials in animals treated for BRD

| Trial | TxGroup | N | *MH*_Arr_  n (%) | MDR_Arr_  n (%) | *MH*_WK3_  n (%) | MDR_WK3_  n (%)** | *MH*_WK10_  n (%) | MDR_WK10_  n (%) |
| --- | --- | --- | --- | --- | --- | --- | --- | --- |
| Fall 2019 | META  NO META  All | 9  9  18 | 3 (33)  1 (11)  4 (22) | 0 (0)  0 (0)  0 (0) | 1 (11)  1 (11)  2 (11)*^a^* | 0 (0)  0 (0)  0 (0)*^d^* | 0 (0)  1 (11)  1 (5.5) | 0 (0)  0 (0)  0 (0) |
| Fall 2020 | META  NO META  All | 7  10  17 | 1 (14)  2 (20)  3 (17) | 0 (0)  0 (0)  0 (0) | 5 (71)  4 (40)  9 (53)^b^*^c^* | 5 (71)  1 (10)  6 (35)*^e^* | 2 (29)  0 (0)  2 (12) | 2 (29)  0 (0)  2 (12) |
| Spring 2021 | META  NO META  All | 4  17  21 | 1 (25)  4 (24)  5 (24) | 0 (0)  0 (0)  0 (0) | 2 (50)  2 (12)  4 (19)*^ab^* | 2 (50)  1 (5.9)  3 (14)*^de^* | 0 (0)  1 (5.9)  1 (4.8) | 0 (0)  0 (0)  0 (0) |
| Fall 2021 | META  NO META  All | 4  14  18 | 0 (0)  8 (57)  8 (44) | 0 (0)  0 (0)  5 (28) | 2 (50)  10 (71)  12 (67)*^c^* | 2 (50)  7 (50)  9 (50)*^e^* | 0 (0)  0 (0)  0 (0) | 0 (0)  0 (0)  0 (0) |
| Overall | META  NO META  All | 24  50  74 | 5 (21)  15 (30)  20 (27) | 0 (0)  5 (10)  5 (6.8) | 10 (42)  17 (34)  27 (36) | 9 (38)  9 (18)  18 (24) | 2 (8.3)  2 (4)  4 (5.4) | 2 (8.3)  0 (0)  2 (2.7) |

**Legend:** An asterisk (*) in the ‘Overall’ row represents that there was an overall statistical difference between META and NO META TxGroups (χ^2^ test, P<0.05). A double asterisk (**) in the header indicates there was a statistically significant difference between a measurement compared to arrival (McNemar’s χ^2^ test, *P*=0.0022). Values with different superscripts in the same column of the ‘All’ rows represent a statistically significant difference among trials (*abc*, *de* Pairwise Fisher’s Exact test with Benjamini-Hochberg adjustment, *P*<0.05). Abbreviations: *MH*, *Mannheimia haemolytica*; MDR, multi-drug resistant; Arr, arrival; WK3, week 3; WK10, week 10; N, number of animals sampled; META, tulathromycin metaphylaxis; NO META, no tulathromycin metaphylaxis; TxGroup, treatment group. Percentage of *MH* Isolated is based on total animals sampled. Percentage of MDR is based on total animals sampled.
