# Supplementary material for: Tulathromycin metaphylaxis increases nasopharyngeal isolation of multidrug resistant Mannheimia haemolytica in stocker heifers
Source: Front Vet Sci. 2023 Nov 20;10:1256997. doi: 10.3389/fvets.2023.1256997 (PMC10694364; doi:10.3389/fvets.2023.1256997)
Supplement: Supplementary file 1 [file Data_Sheet_1.zip › Table S9.docx]

**Table S9.** Resistance gene patterns and association with ICE

| ICE | Number of Isolates  (META/NO META) | Resistance Genes |
| --- | --- | --- |
| No (99) | 95 (37/58) | None |
|  | 4(2/2) | *bla*_ROB_ |
| Yes (148) | 1 (0/1) | None |
|  | 46 (25/21) | *tetH* |
|  | 6 (3/3) | *bla*_OXA_, *bla*_ROB_, *erm(42)*, *msr(E), tetH* |
|  | 10 (7/3) | *bla*_OXA_, *bla*_ROB_, *erm(42)*, *msr(E), tetH,* *tetR* |
|  | 14 (14/1) | *bla*_OXA_, *erm(42)*, *msr(E), tetH,* *tetR* |
|  | 9 (8/1) | *bla*_OXA_, *erm(42)*, *msr(E), tetH* |
|  | 1 (1/0) | *aph(3’’), bla*_OXA_, *erm(42)*, *msr(E), tetH, tetR* |
|  | 16 (16/0) | *aph(6’), bla*_OXA_, *floR, erm(42)*, *msr(E), tetH, tetR* |
|  | 6 (6/1) | *aph(6’), bla*_OXA_, *floR, erm(42)*, *msr(E), tetH, tetR* |
|  | 1(1/0) | *bla*_ROB_, *tetH* |
|  | 1(0/1) | *bla*_ROB_, *msr(E)*, *tetH* |
|  | 15 (8/7) | *aph(6’), bla*_OXA_, *bla*_ROB_, *erm(42)*, *msr(E),floR,tetH* |
|  | 21 (12/9) | *aph(6’), bla*_OXA_, *bla*_ROB_, *erm(42)*, *msr(E),floR, tetH, tetR* |
|  | 1 (1/0) | *aph(6’),bla*_ROB_, *tetH* |

**Legend:** ICE Yes or No indicates that ICE associated genes were found in that Isolate. This table includes all *MH* identified at any time. Abbreviations: ICE, integrative conjugative element; META, tulathromycin metaphylaxis; NO META, no tulathromycin metaphylaxis.
